# Effect of γ-Aminobutyric Acid (GABA) on the Metabolome of Two Strains of *Lasiodiplodia theobromae* Isolated from Grapevine

**DOI:** 10.3390/molecules25173833

**Published:** 2020-08-23

**Authors:** Maria Michela Salvatore, Carina Félix, Fernanda Lima, Vanessa Ferreira, Ana Sofia Duarte, Francesco Salvatore, Artur Alves, Ana Cristina Esteves, Anna Andolfi

**Affiliations:** 1 Department of Chemical Sciences, University of Naples ‘Federico II’, 80126 Naples, Italy; mariamichela.salvatore@unina.it (M.M.S.); frsalvat@unina.it (F.S.); 2 Centre for Environmental and Marine Studies (CESAM), Department of Biology, University of Aveiro, 3810-193 Aveiro, Portugal; carinafelix89@gmail.com (C.F.); nandalima85@gmail.com (F.L.); fvanessa@ua.pt (V.F.); artur.alves@ua.pt (A.A.); 3 Center for Interdisciplinary Research in Health (CIIS), Faculty of Dental Medicine, Universidade Católica Portuguesa, 3504-505 Viseu, Portugal; asduarte@viseu.ucp.pt; 4 BAT Center-Interuniversity Center for Studies on Bioinspired Agro-Environmental Technology, University of Napoli ‘Federico II’, 80138 Naples, Italy

**Keywords:** secondary metabolites, indole-3-carboxylic acid, grapevine, *Lasiodiplodia theobromae*, plant diseases

## Abstract

The effect of γ-aminobutyric acid (GABA) on the metabolome of two strains of *Lasiodiplodia theobromae* isolated from grapevine that hold a different degree of virulence to the host plant (LA-SOL3 (more virulent), LA-SV1 (less virulent)) was investigated. The culture filtrates and crude extracts from the two strains grown in the presence and absence of 10 mM of GABA were tested for phytotoxicity on tomato plant cuttings and leaves, respectively. Considering the opportunistic nature of this fungus for humans, crude extracts were also tested for cytotoxicity on mammalian cell lines. We found that culture filtrates and crude extracts have a decreased toxicity in the presence of GABA. Metabolomic analysis, conducted on both strains at both growth conditions, revealed the production of several compounds, such as indole-3-carboxylic acid (ICA, which is the main compound produced by *L. theobromae*), 3-indolecarboxyaldehyde, (3*R*,4*S*)-botryodiplodin, (*R*)-mellein. Finally, data demonstrate that GABA both induces a decrease in the amount of ICA, and a diversification of the metabolites produced by *L. theobromae*.

## 1. Introduction

In vivo, fungi are challenged by diverse biotic and abiotic stressors (e.g., other organisms, nutrient availability, pH and temperature), which determine several fungal responses. In particular, fungi have developed the capacity to synthesize a plethora of secondary metabolites that are involved in interactions with plants or animals [1]. In fact, during the interaction between fungi and other organisms, secondary metabolites might be involved in very different functions, such as mediating communication within one species or between different species, defence against competitors, nutrient acquisition, and even in symbiotic interactions [2,3].

It is also known that the expression of some fungal genes, including genes required for virulence, is influenced by temperature and nutritional state [4,5]. More recently, new evidences have emerged from studies on the effect of abiotic factors on the metabolome of fungi isolated from several hosts [6]. In fact, different metabolomic profiles have been reported for the fungal pathogen *Lasiodiplodia theobromae* when the strains were grown at different temperatures [7,8].

Within our interest, aiming at understanding the effect of abiotic and biotic factors on fungal secondary metabolites profile and virulence [7,8,9], we assessed the role of γ-aminobutyric acid (GABA) in the toxicity of *L. theobromae*. In fact, GABA might play several roles in plants, ranging from an involvement in nitrogen metabolism, the regulation of cytosolic pH, protection against oxidative stress, and the mediation of interactions between plants and other organisms, such as bacterial and fungal pathogens [10]. Furthermore, levels of GABA in plants are influenced by a number of stressors, including heat, cold, salinity, drought, hypoxia, mechanical damage, and pathogens [11]. Its concentration rises substantially from about 0.8 mM to 2–3 mM during infection, and its level may influence the pathogen responsible for the plant infection. On the other hand, GABA represents a source of nitrogen for fungi, which might be affected by this nutrient availability [12]. Considering the possible involvement of GABA in the interactions between the host and pathogen, fungal secondary metabolite production might be affected by the levels of GABA in plants. Surprisingly, little is known about the effect of GABA on fungal metabolite production. Therefore, the objective of this study was to understand if GABA modulates the virulence of *L. theobromae* via metabolome. We have studied the production of secondary metabolites by two strains of *L. theobromae* [LA-SOL3 (more virulent), LA-SV1 (less virulent)] isolated from grapevine. Hence, metabolites’ profiles obtained under two growth conditions (i.e., 0 mM and 10 mM of GABA) were compared, and the phytotoxicity and cytotoxicity of culture filtrates and organic extracts were evaluated.

## 2. Results

### 2.1. Biomass

**Figure 1:**
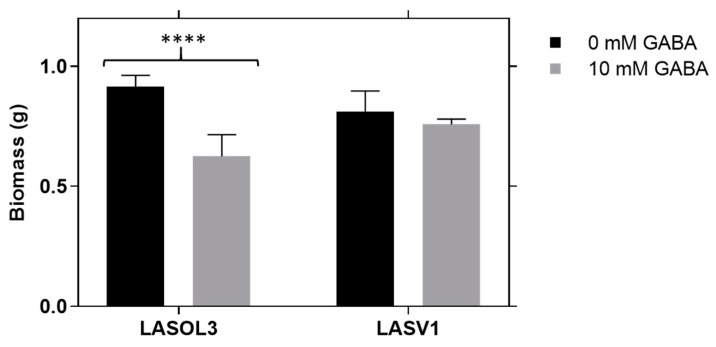
Effect of γ-aminobutyric acid (GABA) on the biomass of *Lasiodiplodia theobromae* (LA-SOL3 and LA-SV1). Data are presented as average ± standard deviation. Statistical analysis was performed using two-way ANOVA, followed by a Tukey multiple comparison test (0 mM GABA/10 mM GABA). Asterisks correspond to data, with statistical difference (**** *p* < 0.0001) between the same isolates; without an asterisk, there is no significant difference.

*Lasiodiplodia theobromae* biomass was determined in liquid medium (Figure 1) [13]. The biomass growth profiles of strains LA-SOL3 and LA-SV1 are similar. In fact, both strains exhibit a biomass decrease in the presence of 10 mM of GABA.

### 2.2. Phytotoxicity Assays

Phytotoxicity of culture filtrates from the two strains of *L. theobromae*, LA-SOL3 and LA-SV1, grown in the presence and absence of 10 mM of GABA, was evaluated by an assay on tomato stems of rootless plants (Table 1).

**Figure 2:**
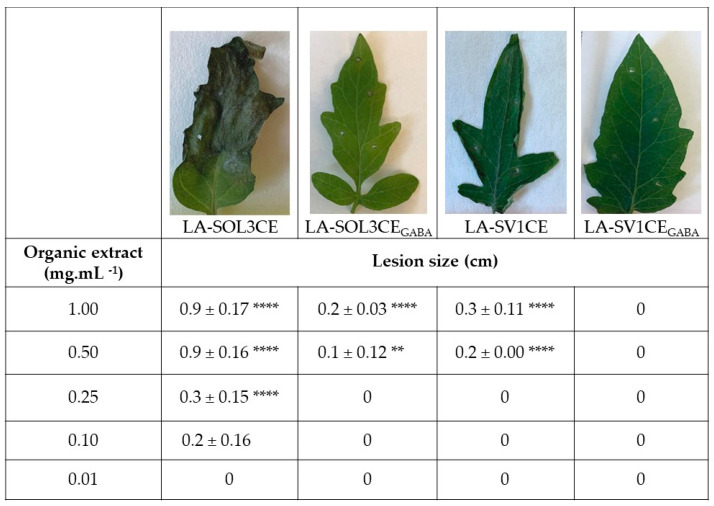
Phytotoxicity of crude extracts (1 mg mL^−1^) of LA-SOL3 and LA-SV1 grown in absence (LA-SOL3CE and LA-SV1CE) and in the presence of γ-aminobutyric acid (GABA) at 10 mM (LA-SOL3CE_GABA_ and LA-SV1CE_GABA_). Statistical analysis was performed using two-way ANOVA, followed by a Tukey multiple comparison test. Asterisks correspond to data with statistical difference (**** *p*< 0.0001, ** *p* < 0.01, when compared to control.

Phytotoxicity tests were also conducted for crude extracts (CE) of culture filtrates, obtained as described in the methods section (called “LA-SOL3CE, LA-SOL3CE_GABA_, LA-SV1CE, LA-SV1CE_GABA_” from this point forward), by leaf puncture assay (Figure 2). The crude extracts of culture filtrates of LA-SOL3 are more phytotoxic for tomato leaves than those obtained from LA-SV1, and, after extraction, GABA seems to decrease their activity.

### 2.3. Cytotoxicity Assays

Cytotoxicity was evaluated for crude extracts obtained from culture of strains LA-SOL3 and LA-SV1, grown in the presence and absence of 10 mM of GABA, to mammalian cell cultures.

Culture filtrates of the strains were not evaluated, because GABA, as neurotransmitter in animals, affects the viability of the cells not allowing the right evaluation of the culture filtrates toxicity [14,15].

**Figure 3:**
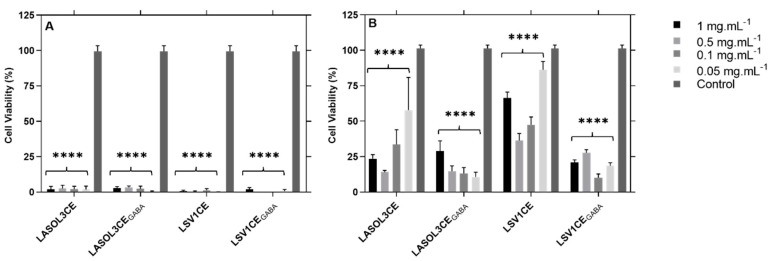
Cytotoxicity of crude extracts from *L. theobromae* LA-SOL3 and LS-V1, grown in the presence (i.e., LA-SOL3CE_GABA_ and LA-SV1CE_GABA_) and absence of 10 mM of γ-aminobutyric acid (i.e., LA-SOL3CE and LA-SV1CE) in 3T3 cells (**A**) and Vero cells (**B**). Phosphate Buffered Saline (PBS) was used as control. Statistical analysis was performed using two-way ANOVA, followed by a Tukey multiple comparison test. Asterisks correspond to data with statistical difference (**** *p* < 0.0001) to the control.

Both isolates of *L. theobromae* show cytotoxicity (Figure 3) in both cell lines tested (3T3 and Vero Cells), at all concentrations of the crude extracts tested.

### 2.4. Metabolites Identification and Quantification of ICA via GC-MS

Culture filtrates of strains LA-SOL3 and LA-SV1 of *L. theobromae*, grown in the absence and presence of 10 mM GABA, were extracted with ethyl acetate (EtOAc), as described in Materials and Methods. Four crude extracts (CE), identified respectively as “LA-SOL3CE, LA-SOL3CE_GABA_, LA-SV1CE and LA-SV1CE_GABA_”, were obtained.

**Figure 4:**
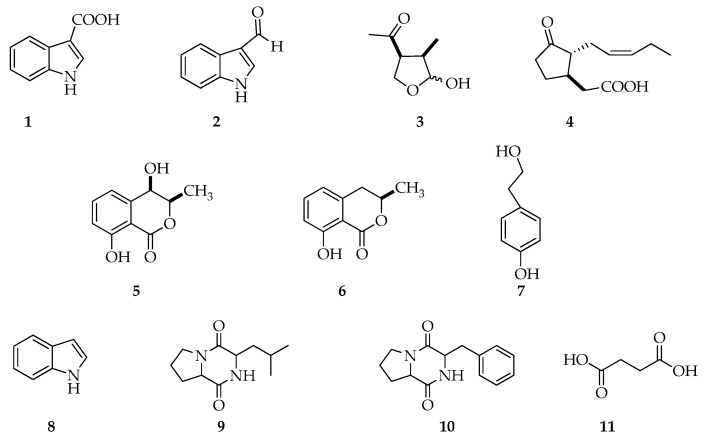
Structures of indole-3-carboxylic acid (**1**), 3-indolecarboxaldehyde (**2**), (3*R*,4*S*)-botryodiplodin (**3**), (−)-jasmonic acid (**4**), (3*R*,4*R*)-4-hydroxymellein (**5**), (*R*)-mellein (**6**), tyrosol (**7**), indole (**8**), *cyclo*-(Pro-Leu) (**9**), *cyclo*-(Pro-Phe) (**10**), succinic acid (**11**), compounds produced by *Lasiodiplodia theobromae* LA-SOL3 and LA-SV1 grown in the presence (10 mM) and absence of γ-aminobutyric acid (GABA).

From the analysis of crude extracts and their chromatographic fractions, 11 metabolites, presented in Figure 4, were detected and characterized via GC-MS (Figures S1–S8). Furthermore, indole-3-carboxylic acid, (3*R*,4*S*)-botryodiplodin, (3*R*,4*R*)-4-hydoxymellein, (*R*)-mellein were isolated from crude extracts, as reported in Materials and Methods (paragraph 5.4), and their structures and stereostuctures have been further confirmed by spectroscopic analyses (Figures S9–S11) and optical rotations, as shown below.

Secondary metabolites produced by fungi may have different structures and physico-chemical properties. Therefore, in order to perform an accurate screening of detectable compounds produced by the strains under examination, samples were analyzed directly or derivatized before GC-MS injection with *N*,*O*-bis(trimethylsilyl)-trifluoroacetamide (BSTFA) or diazomethane in ether.

In fact, the chemical derivatization of samples may facilitate the GC-MS measurements of some compounds, reducing the polarity of functional groups and increasing the sensitivity [16].

**Figure 5:**
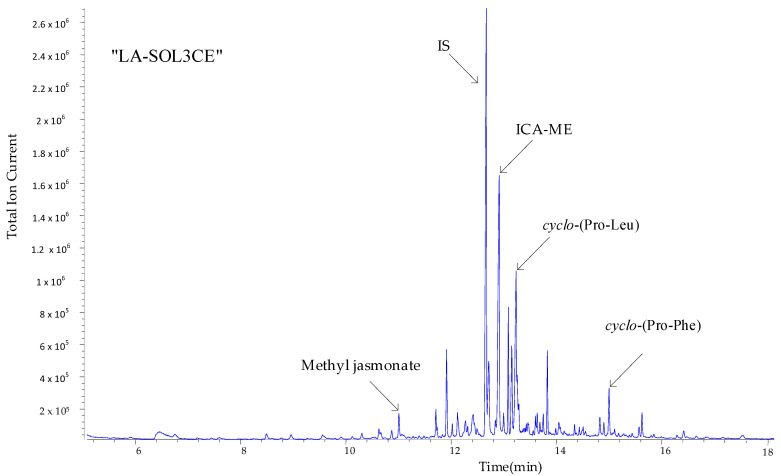
Annotated total ion chromatograms (TICs) acquired by processing crude extract of *Lasiodiplodia theobromae* LA-SOL3 (LA-SOL3CE) after derivatization with diazomethane in ether.

For instance, Figure 5 presents the reconstructed gas chromatogram acquired by presenting to the GC-MS system the “LA-SOL3CE” (after derivatization with diazomethane in ether).

As can be seen, a number of metabolites in Figure 4 are produced in sufficient amounts to be directly detected and identified, via their mass spectra, on the crude extracts.

The pattern in Figure 5 is replicated with minor changes for all four crude extracts (Figures S1 and S2).

A well-developed peak appears in the total ion chromatograms (TIC) of all crude extracts (at retention time ~12.5 min), which is easily attributed, from the associated mass spectrum (Figure S3 (Supplementary Materials)), to indole-3-carboxylic acid methyl derivative (ICA-ME).

On the basis of this fact, ICA appears as the most suitable pilot metabolite to map quantitative differences that might exist in metabolites production when the two *L. theobromae* strains are grown in the presence and absence of 10 mM GABA.

**Figure 6:**
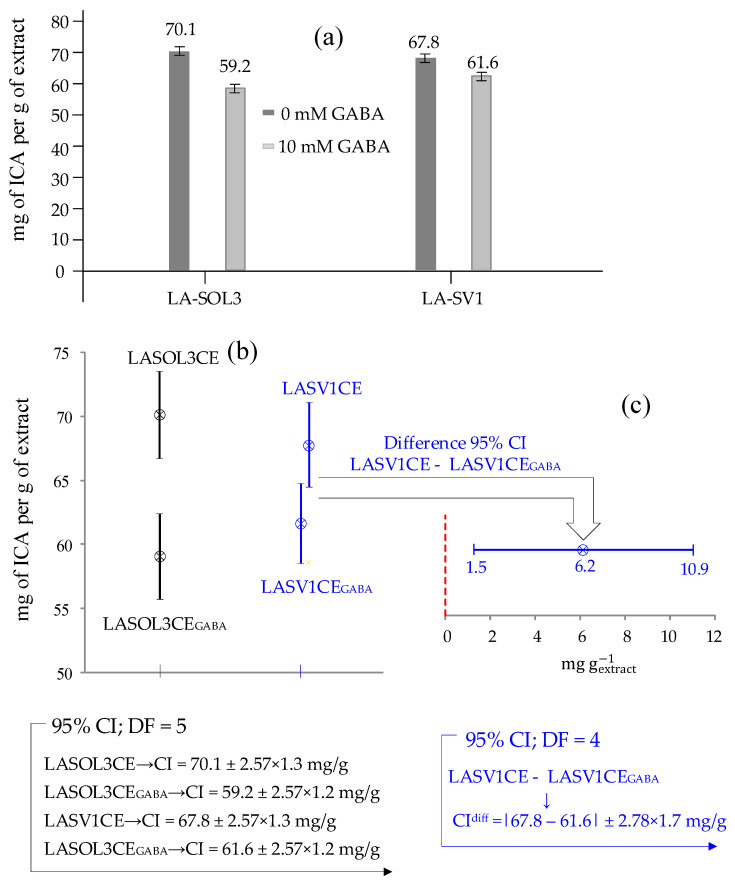
(**a**) Concentrations, C, and standard deviations, $s_{C}$ (expressed as mg per g of extract) of indole-3-carboxylic acid (ICA, **1**) in crude extracts of *Lasiodiplodia theobromae* LA-SOL3 and LA-SV1 grown in the presence (10 mM) and absence of γ-aminobutyric acid (GABA) (i.e., LA-SOL3CE, LA-SOL3CE_GABA_, LA-SV1CE and LA-SV1CE_GABA_). (**b**) Here, 95% Confidence Interval about ICA concentrations in (**a**), and (**c**) 95% CI about the difference of ICA concentrations in LA-SV1CE e LA-SV1CE_GABA_.

Figure 6a shows the indole-3-carboxylic acid (ICA, **1**) concentrations, $C\;\left({\mathrm{mg}\;g}_{\mathrm{extract}}^{-1}\right)$, and the corresponding standard deviations ($s_{C}$) determined in the LA-SOL3CE, LA-SOL3CE_GABA_, LA-SV1CE and LA-SV1CE_GABA_, from which it appears that the production of ICA is decreased in the presence of 10 mM GABA.

In order to state whether differences in concentrations exposed in Figure 6a are statistically significant, a Confidence Interval (CI), at 95% confidence level, has been associated with each of the four concentrations.

To this end, first, for each concentration, the standard deviation, $s_{C}$, shown in Figure 6a, has been calculated using statistics evaluated from the calibration plot, which consists of a regression line through 7 calibration points, corresponding to (7 − 2) = 5 degree of freedom (DF).

Then, the 95% CI is obtained, for each concentration, from relation CI = $C\pm t_{5}\times s_{C}$ ($t_{5}$ = 2.57 represents the so called “*t*-statistic” for 5 DF).

The computed 95% confidence intervals about concentrations exposed in Figure 6a are presented and compared in Figure 6b.

From Figure 6b, it is trivial to deduce that the difference in concentration of ICA observed for LASOL3CE and LASOL3CE_GABA_ is statistically significant at 95% confidence level, simply because the corresponding 95% CI do not overlap.

From this, it is not necessary to conclude that difference between concentrations is not statistically significant when confidence intervals around them overlap (as in the case of 95% CI about ICA concentrations in LASVCE1 and LASV1CE_GABA_, see Figure 6b).

In fact, the difference might still be significant, even when there is some overlap between confidence intervals, and this is just the case of LASV1CE and LASV1CE_GABA_.

This is shown in a more direct way in Figure 6c, which presents the 95% confidence interval (CI^diff^) about the difference (67.8 − 61.6) = 6.2 ${\mathrm{mg}\;g}_{\mathrm{extract}}^{-1}$ between concentrations of ICA in LASV1CE and LASV1CE_GABA_.

CI^diff^ has been evaluated by the formula CI^diff^ = $6.2\pm t_{4}\times s_{\mathrm{diff}}$ (in which the “*t*-statistic” for 4 degree of freedom is used, because one degree of freedom is lost in taking the difference between the two concentrations), and $s_{\mathrm{diff}}$ is the difference standard deviation (which is easily calculated from standard deviations of subtracted individual concentrations (e.g., $s_{C1}$ and $s_{C2}$) through a standard uncertainty propagation formula: $s_{\mathrm{diff}}$ = $\sqrt{s_{C1}^{2}+s_{C2}^{2}}$).

From Figure 6c, at a glance, we can tell that, despite overlapping CI, the difference (between ICA concentrations in LASV1CE and LASV1CE_GABA_) is significant at 95% confidence level simply, because the difference 95% confidence interval does not include zero, which would mean no statistical difference between concentrations.

Apart from that, a number of minor metabolites, reported in Figure 4, were not detectable directly in the crude extracts via GC-MS, but they were identified in fractions of the crude extracts, purified via column chromatography on silica gel, both using GC-MS and NMR spectroscopy.

**Figure 7:**
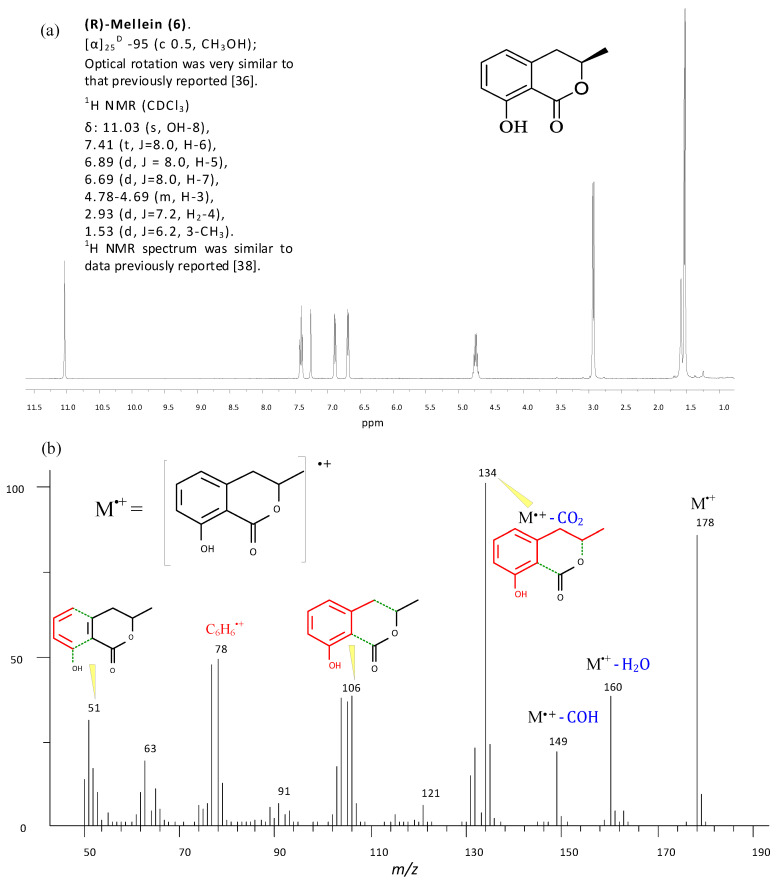
(**a**) ^1^H NMR spectrum of (*R*)-mellein (**6**) recorded at 400 MHz in CDCl_3_; (**b**) 70 eV EI mass spectrum of mellein (ionic fragments are colored in red and neutral losses from the molecular ion, M^+^, are colored in blue).

For instance, (*R*)-mellein (**6**) has been identified and structurally characterized, in CC purified fractions of extracts, from its ^1^H NMR presented in Figure 7a, and from its 70 eV EI mass spectrum in Figure 7b.

Additional NMR and MS data for the identified metabolites in Figure 4 are provided in the Supplementary Materials.

The stereostructure elucidation of some compounds [i.e., (3*R*,4*S*)-botryodiplodin, (3*R*,4*R*)-4-hydroxymellein and (*R*)-mellein] were performed by comparing the ^1^H NMR spectra and optical rotation (OR) of pure compounds, obtained after purification processes, with those reported in literature [8].

Finally, the occurrence of metabolites when the fungi was grown in the presence and in absence of 10 mM of GABA is shown in Table 2.

Altogether, data suggest that GABA both induces a decrease in the amount of indole-3-carboxylic acid (ICA, **1**), and a diversification of the metabolites produced by *L. theobromae*.

## 3. Discussion

*Lasiodiplodia theobromae* is responsible for severe damages in many cultivated and wild host plants worldwide [17,18]. It can act as a primary or secondary pathogen, or as an endophyte that, under stress conditions to the host plant, can become pathogenic [19]. *L. theobromae* has also been reported as an “emerging pathogen” in human, causing infections, especially in immunocompromised patients [7,20,21,22].

The increasing economic importance of plant diseases caused by *L. theobromae* led us to speculate that secondary metabolites production might be affected by abiotic factors.

Within our recent activity, the findings on the study of effect of temperature on metabolite production of *L. theobromae* strains have been reported [7,8].

In the present study, *L. theobromae* strains LA-SOL3 and LA-SV1 isolated from grapevine in Peru, and already investigated for secondary metabolites production in Czapek medium at two different temperatures (i.e., 25 °C and 37 °C) [8], were grown in potato dextrose broth (PDB), in the presence and absence of 10 mM of γ-aminobutyric acid (GABA). Hence, the aim of this study was to compare the metabolite’s profile obtained when the strains were grown in PDB, with the one obtained in Czapek medium [8], and also to evaluate the effect of GABA on the secondary metabolite production, which is related to the toxicity of culture filtrates and organic extracts.

*L. theobromae* strains exhibit a similar biomass growth profile, showing lower biomass values in the presence of 10 mM of GABA.

When these strains were grown in Czapek medium at 25 °C, the metabolite profile was distinct from the one obtained in PDB at the same growth temperature. In fact, metabolites such as (3*S*,4*R*,5*R*)-4-hydroxymethyl-3,5-dimethyldihydro-2-furanone, (3*R*,4*S*)-4-acetyl-3-methyl-2-dihydrofuranone, lasiolactols A-B were produced only in Czapek medium [8], while metabolites, such as 3-indolecarboxaldehyde (**2**), *cyclo*-(Pro-Phe) (**9**), *cyclo*-(Leu-Pro) (**10**), were produced only in PDB. Hence, the first evidence is that nutrients availability affects the production of secondary metabolites.

The detection of ICA in crude extracts turns out to be particularly relevant, because many *L. theobromae* strains, associated with different hosts [7,8], produce this metabolite. In fact, crude extracts from culture filtrates of grapevine-isolated strains grown in PDB produce (**1**) as a main compound. The quantification of (ICA, **1**) in crude extracts of LA-SOL3 and LA-SV1 grown in the presence and absence of 10 mM of GABA shows the decrease of (**1**) production when both strains were exposed to GABA. It is justified because (**1**) is a phytotoxic compound involved in the fungal infections. Several *L. theobromae* strains produce phytohormones, in particular indole derivatives and jasmonates [6]. Indole-3-carboxylic acid is also considered a phytohormone, commonly associated with plants and fungi, and the physiological effects of phytohormones are dose-dependent [23,24]. The biological role of indole-3-carboxylic acid has long been neglected, but some studies have highlighted its potential role in plant as phytoalexin. In fact, **1** has been identified as mediator of induced resistance in Arabidopsis against plant pathogens [25]. Hence, GABA is produced by the plants as defence agent, and reduces the fungal pathogenic activity influencing the production of ICA. In fact, culture filtrates and their extracts obtained from the fungal exposure to GABA are less phytotoxic compared to the ones obtained in the absence of GABA. As reported in Table 2, fungal strains produce several metabolites, and the activity of culture filtrates and extracts is the result of the synergistic effect of indole-3-carboxylic acid and the less represented metabolites.

As has been indicated in other studies [11,26,27,28], the concentration of GABA substantially and rapidly increases the defense responses of the plant in both abiotic and biotic stress conditions. In addition, it has also been shown that GABA is capable of potentiating the synthesis of ethylene in some plasmas (as sunflower), promoting the accumulation of transcripts of ICA [29]. Thus, it is possible to conclude that the addition of GABA was able to reduce the pathogenicity of fungi.

Yuan et al. [30] indicate that salicylic acid (SA), indole-3-acetic acid (IAA) and γ-amino butyric acid (GABA), present cross-signalling networks for plant defence against the phytopathogen *Agrobacterium*, which corroborates that both strains showed a decrease of indole-3-carboxylic acid when the fungi were grown in the presence of GABA, and it was also possible to observe a change in metabolic profile.

## 4. Conclusions

Fungi are recognized as a rich source of secondary metabolites, whose roles in the natural habitat likely include chemical signalling, defence against other microorganisms, and the establishment of symbiosis with host plant. Abiotic factors (e.g., temperature, nutrient availability) are thought to shape the secondary metabolite profiles of fungi, leading to relevant implications in fungal pathogenicity. In fact, the metabolite profile of grapevine strains of *Lasiodiplodia theobromae* changes significantly with the fungal exposure to GABA. We observed a decrease of phytotoxic and cytotoxic activities of culture filtrates and crude extracts, when *L. theobromae* was grown in the presence of GABA. Apparently, these biological data may be related to the decrease of the indole-3-carboxylic acid production when both strains were exposed to GABA. Our findings reveal that GABA influences the virulence of *L. theobromae*, leading to relevant effects on plant-fungus interactions.

## 5. Materials and Methods

### 5.1. General Experimental Procedures

Optical rotations of pure metabolites were measured in CHCl_3_ or in MeOH on a Jasco polarimeter (Tokyo, Japan). ^1^H and ^13^C NMR spectra were recorded at 400 and 100 MHz, respectively, in deuterated chloroform (CDCl_3_) or in deuterated methanol (CD_3_OD), on a Bruker AV400 Spectrometer (Karlsruhe, Germany), and the same solvents were used as internal standards. Analytical TLC were performed on silica gel plates (Kieselgel 60, F254, 0.25, Merck, Darmstadt, Germany). The spots were visualized by exposure to UV radiation (253 nm), or by spraying first with 10% H_2_SO_4_ in methanol, followed by heating at 110 °C for 10 min. Chromatography was performed on silica gel column (Merck, Kieselgel 60, 0.063–0.200 mm).

GC-MS analyses were performed on an Agilent 6850 GC (Milan, Italy), equipped with an HP-5MS capillary column (5% phenyl methyl poly siloxane stationary phase), coupled to an Agilent 5973 Inert MS detector, which was operated in electron ionization (EI) mode at 70 eV and at 200 °C. Helium was used as carrier gas, set at 1 mL·min^−1^. The injector temperature was 250 °C and the temperature ramp raised the column temperature from 70 °C to 280 °C: 70 °C for 1 min; 10 °C·min^−1^ until reaching 170 °C; and 30 °C·min^−1^ until reaching 280 °C. Then, it was held at 280 °C for 5 min. The quadrupole mass filter was kept at 250 °C, and mass spectra were scanned from mass-to-charge ratio (*m*/*z*) 45–550, at a frequency of 3.9 Hz.

### 5.2. Fungal Strains and Growth Conditions

*Lasiodiplodia theobromae* strains LA-SOL3 and LA-SV1 used in this study were originally isolated from grapevines in Peru [31]. Cultures were maintained at room temperature on potato dextrose agar (PDA) medium (Merck, Germany). Before inoculations, each strain was cultured on PDA at 25 °C for 4 days. Mycelium was scraped from PDA plates and suspended in 5 mL of sterilized ultra-pure water. This mycelial suspension was added to 1 L Erlenmeyers, containing 250 mL of potato dextrose broth (PDB, pH 5.6), with 0 mM and 10 mM of GABA, and incubated for 4 days in the dark at 25 °C. Culture filtrates were obtained by filtering the culture through sterile 0.45 µm cellulose membranes in a vacuum system.

### 5.3. Biomass

Four plugs of 5 mm diameter from actively growing cultures of *L. theobromae* LA-SOL3 and LA-SV1 on PDA were inoculated on 250 mL of PDB in 1 L Erlenmeyers, in the presence and absence of 10 mM of GABA, and incubated as reported in the previous subparagraph. After the incubation period, the mycelium was separated from the culture medium by filtration (filter paper). The mycelium was dried at 50 °C for 48 h and the dry weight determined [13].

### 5.4. Extraction and Purification of Fungal Metabolites

Culture filtrates of *L. theobromae* LA-SOL3 and LA-SV1, grown in the presence and absence of 10 mM of GABA (i.e., LA-SOL3 = 910 mL, LA-SOL3_GABA_ = 950 mL, LA-SV1 = 900 mL, LA-SV1_GABA_ = 950 mL), were extracted 3 times with ethyl acetate (EtOAc). Each crude extract (CE) was dried with Na_2_SO_4_, and evaporated under reduced pressure originating as a brown oil residue (i.e., LA-SOL3CE = 113.7 mg, LA-SOL3CE_GABA_ = 84.7 mg, LA-SV1CE = 114.7 mg, LA-SV1CE_GABA_ = 76.1 mg). These extracts were purified by column chromatography (CC) on silica gel producing the identified metabolites (**1–11**).

LA-SOL3CE was fractionated by CC on silica gel (40 cm × 1.5 cm), eluted with CHCl_3_/*i*-PrOH (9:1, *v*/*v*) originating 10 homogeneous fractions, and the last fraction was eluted with methanol (A 3.7 mg, B 2.2 mg, C 10.3 mg, D 10.4 mg, E 10.2 mg, F 9.1 mg, G 9.9 mg, H 10.6 mg, I 8.2 mg, L 26.6 mg). Fraction B was identified as (*R*)-mellein and fraction D was identified as (3*R*,4*S*)-botryodiplodin. Fraction G was identified as indole-3-carboxylic acid.

LA-SOL3CE_GABA_ was fractionated by CC on silica gel (40 cm × 1.5 cm) eluted with CHCl_3_/*i*-PrOH (9:1, *v*/*v*) originating 11 homogeneous fractions, and the last fraction was eluted with methanol (A 1.5 mg, B 1.1 mg, C 2.2 mg, D 1.4 mg, E 4.9 mg, F 4.1 mg, G 4.0 mg, H 6.2 mg, I 23.2 mg, L 6.4, M 21.0 mg). Fraction A was identified as (*R*)-mellein, fraction D was identified as *cis*-4-hydroxymellein and fraction F was identified as (3*R*,4*S*)-botryodiplodin.

LA-SV1CE was fractionated by CC on silica gel (40 cm × 1.5 cm) eluted with CHCl_3_/*i*-PrOH (9:1, *v*/*v*), originating 10 homogeneous fractions and the last fraction was eluted with methanol (A 1.7 mg, B 1.8 mg, C 4.1 mg, D 4.4 mg, E 1.8 mg, F 7.9 mg, G 0.8 mg, H 2.3 mg, I 14.0 mg, L 19.5 mg). Fraction B was identified as (*R*)-mellein, and I was identified as indole-3-carboxylic acid.

LA-SV1CE_GABA_ was fractionated by CC on silica gel (40 cm × 1.5 cm) eluted with CHCl_3_/*i*-PrOH (9:1, *v*/*v*) originating 11 homogeneous fractions and last fraction was eluted with methanol (A 1.3 mg, B 2.0 mg, C 4.4 mg, D 3.8 mg, E 5.1 mg, F 6.3 mg, G 3.6 mg, H 1.7 mg, I 6.2 mg, L 5.2 mg, M 10.3 mg). Fraction E was identified as (3*R*,4*S*)-botryodiplodin.

### 5.5. Metabolites Identification

In order to identify bioactive compounds that may be involved in the biological activities of the culture filtrates of two strains, their crude extracts (LA-SOL3CE, LA-SOLCE_GABA_, LA-SV1CE, LA-SV1CE_GABA_) were analysed via GC-MS (see Figure 5, Figures S1 and S2 (Supplementary Materials)).

Some minor metabolites in Figure 4 were not detectable directly in the crude extracts via GC-MS, but they were identified by analyzing the chromatographic fractions of the crude extracts (see previous section) (see Supplementary Materials for MS and ^1^H NMR spectra).

Crude extracts or chromatographic fractions were analysed directly, after trimethylsilylation with *N*,*O*-bis (trimethylsilyl)-trifluoroacetamide (BSTFA) (Fluka, Buchs, Switzerland), and after treatment with diazomethane in ether, as described in [16,32,33].

Metabolites identification was conducted comparing EI mass spectra at 70 eV with spectra of known substances present in the NIST 14 mass spectral library [34], or in the custom GC-MS library described by Félix et al. [7,8]

In general, identification via MS libraries search was further supported by Kovats retention index (RI), attributed to each analyte from the chromatographic data and by checking the quality of mass spectra used for identification, by using an ad hoc Windows application (i.e., QC_Soft), developed in our MS laboratory (Naples, Italy) within the MATLAB/GUIDE development environment (MathWorks Inc., Natick, MA, USA) [35].

Confidence in the identification of metabolites via GC-MS is fundamentally based on the ability to acquire the mass spectra of the pure substance to be identified. This condition becomes increasingly difficult to fulfill as the complexity of the injected sample increases (because of components coelution) and the absolute amount of sought components in the mixture decreases (because of mass spectra contamination by background signals).

However, detection of small amounts of components in complex mixtures can be challenged in various ways.

The deconvolution of GC-MS data is a useful strategy (implemented in a number of ad hoc applications, e.g., NIST/AMDIS [36]), to remedy difficulties created by components coelution, and which, in not cases which are not that severe, proves to be successful.

Analogously, the derivatization of target components to be identified can be thought as producing effects similar to the increase of the effective amount of an analyte presented to the GC-MS.

In order to extend the range of detected analytes, deconvolution and derivatization can be complemented by the strategy of simplifying the composition of injected mixtures, and of actually increasing the amount of components to be identified.

This strategy has been implemented in this study by subjecting crude extracts to preliminary on column separations (see previous section). Ensuing chromatographic fractions, which are simpler and contain higher amounts of minor components, are then presented to the GC-MS system, so that, presumably, the mass spectra of pure components can be acquired.

Obviously, it is very difficult or impossible to state, by simple inspection, if a given mass spectrum is due to a single component or it contains extraneous mass peaks which will hinder identification. However, this dilemma can be solved a posteriori, i.e., after a substance has been putatively associated to the mass spectrum.

**Figure 8:**
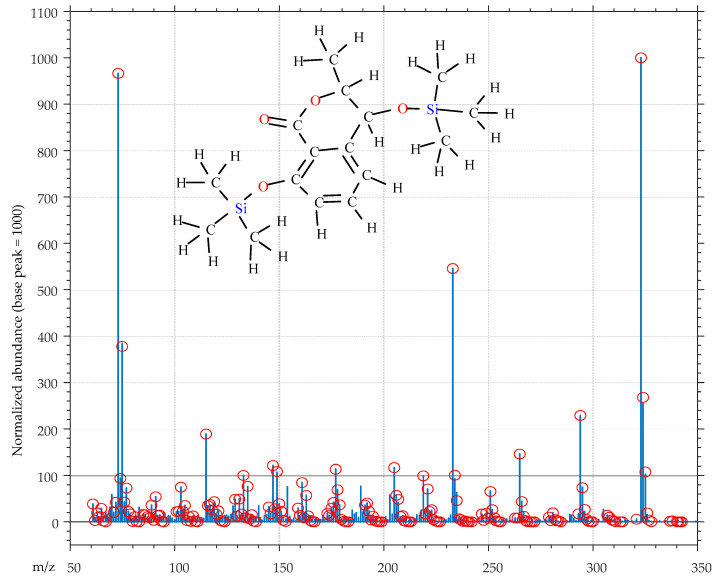
Ex post quality control of mass spectrum of 2TMS *cis*-4-hydroxymellein using the QC_Soft application. Mass peaks marked with red circles are associated to fragments created, by the software algorithm, from the hypothesized structure.

For instance, consider the normalized mass spectrum in Figure 8, and suppose that we wish to state the quality of this mass spectrum for identification purposes.

We see that, on the *m*/*z* range 60–350, the mass spectrum of Figure 8 exposes 280 mass peaks (almost a peak at each integer *m*/*z* value), which make up a total ionic current of 8604 (the average intensity of a mass peak amounts only to 31 on a scale between 0 and 1000). These data seem to indicate that the spectrum is heavily contaminated by small intensity mass peaks, which is just the situation we attempted to avoid.

However, when this mass spectrum is presented to our QC_Soft application with the structure of 2TMS *cis*-4-hydroxymellein, tentatively associated to it, it results in being a mass spectrum of high quality essentially produced by a single compound.

In fact, Figure 8 shows that most of the mass peaks (making up 84% of the total ionic current of the spectrum) can be associated with fragments which QC_Soft creates from the structure of 2TMS (3*R*,4*R*)-4-hydroxymellein (**5**) (explained mass peaks are marked with red circles in Figure 8).

**5** (C_16_H_2_6O_4_Si_2_, monoisotopic mass 338.1370 u) is a rather complex molecule, with 48 atoms, 45 single bonds and 4 double bonds. From these fundamental structural data, the software algorithm easily determines that the submitted structure contains (49 − 48 +1) = 2 cycles and sets to 3 the maximum number of single bonds which can be broken to create fragments (double bonds of the aromatic ring and carbonyl group are preserved). Contextually, a number of fragmentation rules are selected, and applied during the fragment creation process, by mapping the functional groups in the submitted structure. After this, created fragments are assigned on the basis of their monoisotopic mass, to corresponding peaks the mass spectrum. Finally, fragments, whose composition produces isotopic peaks which are at variance with peaks in the mass spectrum which follow the monoisotopic peak, are filtered out.

Although ex post, Figure 8 is proof that we have succeeded in presenting to the mass spectrometer a sample of 2TMS *cis*-4-hydroxymellein of adequate purity and in sufficient amount for confident identification.

The structure and stereostructure of (3*R*,4*S*)-botryodiplodin, *(3R*,4*R)*-4-hydroxymellein and (*R*)-mellein were confirmed, comparing the ^1^H NMR spectra and optical rotation (OR) of pure compounds, obtained after purification processes, with those reported in literature [8,37,38,39,40].

A summary of the GC-MS and ^1^H NMR data is presented in Supplementary Materials.

### 5.6. Quantification of Indole-3-Carboxylic Acid (ICA)

Certified standard of indole-3-carboxylic acid (Sigma-Aldrich, St. Louis, MO, USA) and weighted amounts of crude extracts were treated with diazomethane in ether, in order to obtain methyl derivative of indole-3-carboxylic acid (ICA-ME) [33].

Solutions of methyl indole-3-carboxylate were prepared in chloroform, at a concentration range of 50–200 mg·L^−1^, and analysed via GC-MS.

The Internal Standard Calibration Method (ISTD) has been employed to quantify ICA-ME. In this respect, the stock ICA-ME solution was diluted with chloroform, to prepare 7 calibration solutions containing accurately known concentrations of the target analyte. Furthermore, each calibration solution was made with 100 mg·L^−1^ of anthracene internal standard (IS) (Sigma-Aldrich, St. Louis, MO, USA).

The calibration plot was made by reporting (at each calibration level) the area under the analyte chromatographic peak, normalized with respect to the area of the internal standard peak, as a function of the known concentration of the analyte expressed as mg·L^−1^. The signal of a reagent blank containing only the IS in chloroform was also measured as control during calibration.

A linear dependence of the normalized signal from the concentration can be seen by a visual inspection of the calibration plot.

In fact, calibration data can be excellently interpolated (R^2^ = 0.997), with a least square regression line (*y* = *a* + *bx*), in which *a* represents the intercept and *b* the slope (L·mg^−1^) of the least square line through the calibration points.

As can be seen from Table 3, from the calibration plot, comprised of 7 calibration points, the following statistics were readily evaluated using standard statistical procedures [41].

(1) The intercept, a, and slope, b, of the regression line through the calibration points and the corresponding standard deviations (respectively, $s_{a}$ and $s_{b}$).

(2) The standard deviation, $s_{y/x}$, of the signal about the regression line. $s_{y/x}$ is a statistic called “residual deviation about the regression”, and is readily obtained from calibration data under the assumption of homoscedastic data [42].

The intercept and slope of the regression line are used to obtain the concentration of ICA, $C^{\prime }$ (mg·L^−1^), in solutions presented to the GC-MS.

The residual deviation about the regression, $s_{y/x}$, is used to evaluate the standard deviation, $s_{C^{\prime }}$ (mg·L^−1^), of the ICA $C^{\prime }$ concentrations. Finally, $C^{\prime }$ concentrations and their standard deviations, $s_{C^{\prime }}$, are converted to the corresponding concentrations and standard deviations in crude extracts of *L. theobromae* LA-SOL3 and LA-SV1, grown in the presence and absence of 10 mM of GABA, were exposed in Figure 6 (i.e., C and $s_{C}$ expressed as mg ICA per g of extract).

This conversion is performed by multiplying $C^{\prime }$ and $s_{C^{\prime }}$ for a coefficient evaluated from the procedure through which the ICA solutions, which were finally presented to the GC-MS, were made up, starting from the crude extracts.

The standard deviation of the intercept, $s_{a}$, is used to calculate the method detection limit (LOD), according to the formula: LOD = $3.3\times s_{a}/b$; and the quantification limit (LOQ), according to the formula: LOQ = $10\times s_{a}/b$ [43].

### 5.7. Phytotoxicity Assay

Phytotoxicity was assessed on tomato cuttings and on tomato leaves. Phytotoxicity of culture filtrates of strains LA-SOL3 and LA-SV1, diluted in distilled sterile water, were tested on tomato cuttings (1:4, 1:2 (*v*/*v*) and without dilution). Tomato stems of rootless plants (2 weeks old) were dipped in vials with 2 mL of culture filtrate. After incubation (24 h) at room temperature, the solution was replaced by 2 mL of sterile distilled water for an additional 48 h. Sterile distilled water, PDB medium and 10 mM solution of GABA were used as controls. Using a 0–4 scale (0: no symptoms; 1: slight withering; 2: intermediate withering; 3: severe withering; 4: full withering), symptoms were recorded and converted to a percentage to evaluate the phytotoxic activity [7]. All assays were conducted in triplicate.

Young tomato plant leaves punctured with a sterile needle were used for the leaf puncture assay. Crude extracts were dissolved in methanol, and then a stock solution (4% methanol) was made with distilled water. A droplet (20 µL; 1.00, 0.50, 0.25, 0.10, 0.01 mg·mL^−1^) of the crude extract solution was applied on the adaxial surface of leaves. As control, a droplet of 4% methanol was used. The leaves were kept in moist chambers to prevent the droplets from drying, and were observed daily. The visual symptoms were recorded after 10 days of incubation, and the lesion diameter was expressed in cm [7]. All assays were carried out in triplicate.

### 5.8. Cytotoxicity Assay on Mammalian Cell Lines

In vitro cytotoxicity was performed as previously described [13,44], with slight modifications. Two cell lines were grown and maintained according to Ammerman et al. [45]: a Vero cell line (ECACC 88020401, African Green Monkey Kidney cells, GMK clone) and a 3T3 cell line (ECACC 86110401, mouse embryonic fibroblasts, A31 clone A31). The microtiter plates were incubated at 37 °C in 5% CO_2_ for 24 h. Vero and 3T3 cells were treated, for 20 h, with the crude extracts (1:1 in DMEM-Dulbecco’s Modified Eagle Medium). Four different concentrations of each crude extract were analysed (1, 0.5, 0.1 and 0.05 mg·mL^−1^ in phosphate buffered saline (PBS)). After the incubation period, the medium was removed by aspiration and 50 µL of DMEM with 10% resazurin (0.1 mg·mL^−1^ in PBS) was added to each well to assess cell viability. The microtiter plates were incubated at 37 °C in 5% CO_2_ during 3 h. The absorbance was read at 570 and 600 nm wavelengths in a microtiter plate spectrophotometer (Biotek Synergy, Portugal). PBS was used as control.

### 5.9. Statistical Analysis

Two-way analysis of variance (ANOVA), followed by a Tukey’s multiple comparison test, was used to determine the statistical significance of biomass, phytotoxicity and cytotoxicity between each dilution/concentration and control of each crude extract and culture filtrate obtained from different sources (LASOL3CE, LASOL3CE_GABA_, LASV1CE, LASV1CE_GABA_) (*p* < 0.05, *p* < 0.01, *p* < 0.001, *p* < 0.0001). All the analyses were performed with GraphPad Prism 6 (GraphPad Software, La Jolla, CA, USA). Data are shown as the average of three independent replicates of each condition.

## Figures and Tables

**Table 1:** Phytotoxicity (%) of culture filtrates of LA-SOL3 and LA-SV1 grown in the presence and absence of 10 mM of γ-aminobutyric acid (GABA). Statistical analysis was performed using two-way ANOVA followed by a Tukey multiple comparison test (Water/PDB). Asterisks correspond to data with statistical difference (**** *p* < 0.0001, *** *p* < 0.001) to PDB or H_2_O.

Culture Filtrate	Dilution (*v*/*v*)	Symptoms Level (Mean + SD)	Phytotoxic Activity (%)
Control (PDB)	-	0.7 ± 0.58	16.7
Control (H_2_O)	-	0	0.0
Control (10 mM GABA)	-	0	0.0
LA-SOL3	25/100	1.0 ± 0.00 ****/-	25.0
	50/100	2.0 ± 0.00 ****/****	50.0
	No dilution	4.0 ± 0.00 ****/****	100.0
LA-SOL3_GABA_	25/100	1.0 ± 0.00 ****/-	25.0
	50/100	1.0 ± 0.00 ****/-	25.0
	No dilution	3.0 ± 0.00 ****/****	75.0
LA-SV1	25/100	1.0 ± 0.00 ****/-	25.0
	50/100	1.7 ± 0.58 ****/***	41.7
	No dilution	2.7 ± 0.58 ****/****	66.7
LA-SV1_GABA_	25/100	1.0 ± 0.00 ****/-	25.0
	50/100	1.0 ± 0.00 ****/-	25.0
	No dilution	3.0 ± 0.00 ****/****	75.0

**Table 2:** Distribution of minor secondary metabolites produced by strains LA-SOL3 and LA-SV1, according to strain and γ-aminobutyric acid (GABA) concentration.

Code	Name	LA-SOL3		LA-SV1	
		0 mM GABA	10 mm GABA	0 mM GABA	10 mm GABA
**2**	3-Indolecarboxaldehyde	+	+	-	-
**3**	(3*R*,4*S*)-Botryodiplodin	+	+	-	+
**4**	Jasmonic acid	+	+	-	+
**5**	(3*R*,4*R*)-4-Hydroxymellein	-	+	-	+
**6**	(*R*)-Mellein	+	+	+	+
**7**	Tyrosol	-	+	+	-
**8**	Indole	-	+	+	+
**9**	*Cyclo*-(Pro-Leu)	+	+	+	+
**10**	*Cyclo*-(Pro-Phe)	+	+	+	+
**11**	Succinic acid	+	-	+	+

**Table 3:** Performance data of the methyl ester of indole-3-carboxylic acid (ICA-ME) quantification method.

**ICA-ME** **(RI = 1535)**	**Intercept** $\left(a\pm s_{a}\right)$	**Slope** $\left(b\pm s_{b}\right)$ $\left(L\cdot mg^{-1}\right)$	$s_{y/x}$	**R^2^**	**LOD (mg L^−1^)**	**LOQ (mg L^−1^)**
	−0.0171 ± 0.0089	0.00604 ± 0.00015	0.0124	0.997	4.9	14.8

## Supplementary Materials

The following are available online at https://www.mdpi.com/1420-3049/25/17/3833/s1, Figures S1 and S2: Total Ion Current Chromatograms of crude extracts of two strains of *L. theobromae*; Figures S3–S8: Annotated 70 eV EI Mass Spectra of metabolites 1–11; Figures S9–S12: 1H NMR spectra of isolated metabolites 1,3,5,6.
