# Supplementary material for: Effect of γ-Aminobutyric Acid (GABA) on the Metabolome of Two Strains of Lasiodiplodia theobromae Isolated from Grapevine
Source: Molecules. 2020 Aug 23;25(17):3833. doi: 10.3390/molecules25173833 (PMC7503889; doi:10.3390/molecules25173833)
Supplement: Supplementary file 1 [file molecules-25-03833-s001.pdf]

Supplementary Materials

Effect of γ-Aminobutyric Acid (GABA) on the Metabolome of Two Strains of *Lasiodiplodia theobromae* Isolated from Grapevine

Maria Michela Salvatore[1], Carina Félix[2], Fernanda Lima[2], Vanessa Ferreira[2], Ana Sofia Duarte[3], Francesco Salvatore[1], Artur Alves[2], Ana Cristina Esteves[3,*] and Anna Andolfi[1,4*]

[1] Department of Chemical Sciences, University of Naples 'Federico II', 80126 Naples, Italy; mariamichela.salvatore@unina.it (M.M.S.); frsalvat@unina.it (F.S.)

[2] Centre for Environmental and Marine Studies (CESAM), Department of Biology, University of Aveiro, 3810-193 Aveiro, Portugal; carinafelix89@gmail.com (C.F.); nandalima85@gmail.com (F.L.); fvanessa@ua.pt (V.F.); artur.alves@ua.pt (A.A.)

[3] Center for Interdisciplinary Research in Health (CIIS), Faculty of Dental Medicine, Universidade Católica Portuguesa, 3504-505 Viseu, Portugal; asduarte@viseu.ucp.pt

[4] BAT Center-Interuniversity Center for Studies on Bioinspired Agro-Environmental Technology, University of Napoli 'Federico II', 80138 Naples, Italy.

* Correspondence: acesteves@viseu.ucp.pt (A.C.E.); andolfi@unina.it (A.A.); Tel.: +39-081-2539179 (A.A.)

Academic Editor: Vincenzo De Feo

Received: 8 July 2020; Accepted: 19 August 2020; Published: date

Summary of Supplementary Materials		
Figure	Contents	Comments
Figure S1, Figure S2	Total Ion Current Chromatograms of crude extracts: LA-SOL3CE, LA-SOL3CE$_{GABA}$, LA-SV1CE and LA-SV1CE$_{GABA}$"	Chromatographic peaks of metabolites produced in sufficient amount to be detected on crude extract are annotated.
Figure S3, Figure S4, Figure S5, Figure S6, Figure S7, Figure S8	Annotated 70 eV EI Mass Spectra of metabolites identified in this study (see Figure 4).<br>Kovats Retention index (RI) is reported in legend to figures.	**M$^{•+}$** represents the molecular ion.<br>**Structures:**<br>Parts of structures coloured in red represent ionic fragments.<br>Parts of structures coloured in black represent dark matter.<br>Dashed lines represent cleaved bonds.<br>**Formulas:**<br>Formulas coloured in blue represent neutral losses from the molecular ion.<br>Formulas in red represent ionic fragments.<br>TMS$^+$ represents the trimethylsilyl $(CH_3)_3$-Si$^+$ ion (m/z = 73).
Figure S9, Figure S10 Figure S11, Figure S12	^{1}H NMR spectra recorded at 400 MHz	3-indol-carboxylic acid (**1**),<br>(-)-botryodiplodin (**3**),<br>(3*R*,4*R*)-4-hydoxymellein (**5**),<br>(*R*)-mellein (**6**).

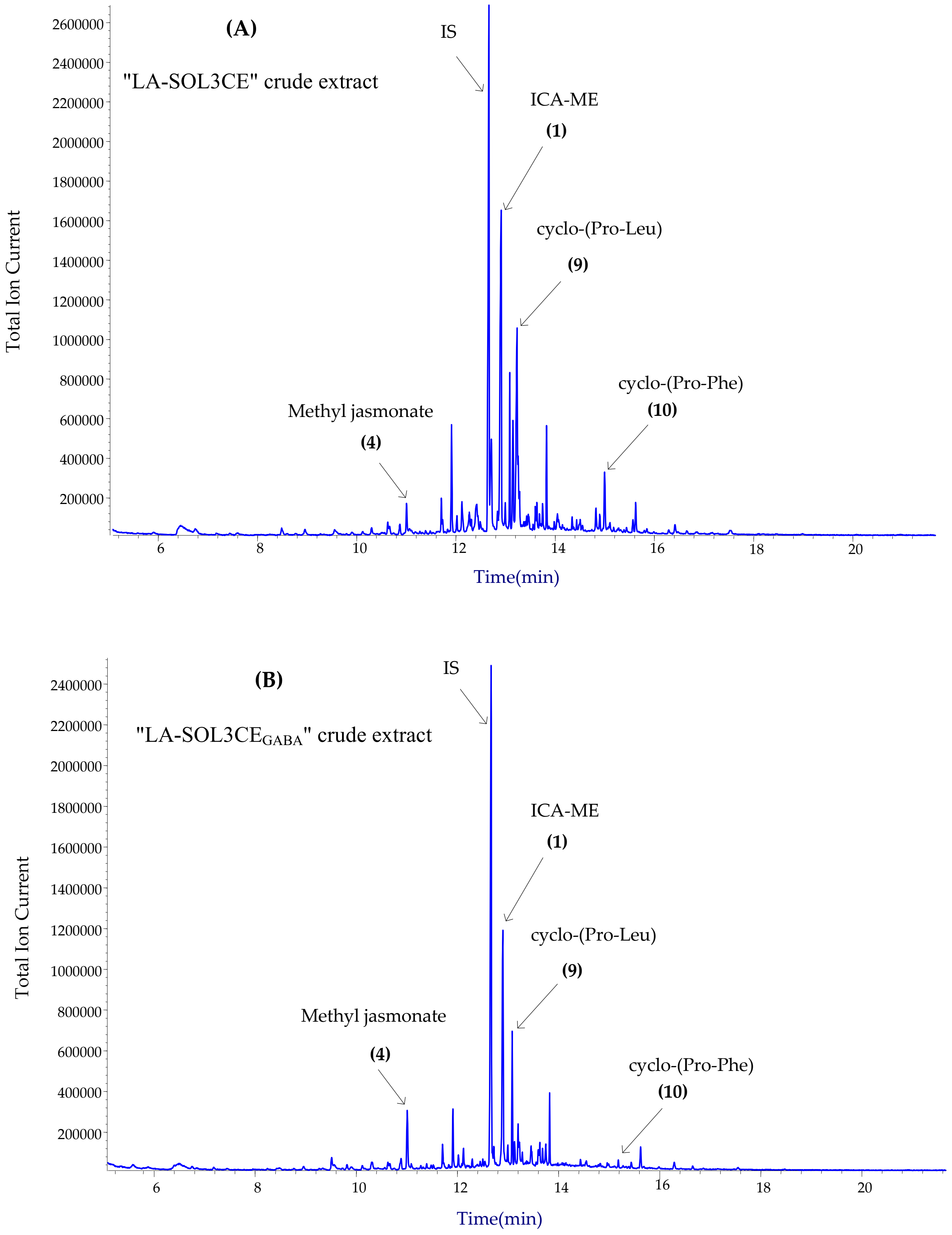

Figure S1. Annotated total ion chromatograms (TICs) acquired by processing crude extracts with diazomethane in ether. (A) Crude extract of *Lasiodiplodia theobromae* LA-SOL3 (LA-SOL3CE); (B) crude extract of *L. theobromae* LA-SOL3 grown in presence of GABA (LA-SOL3CE$_{GABA}$),

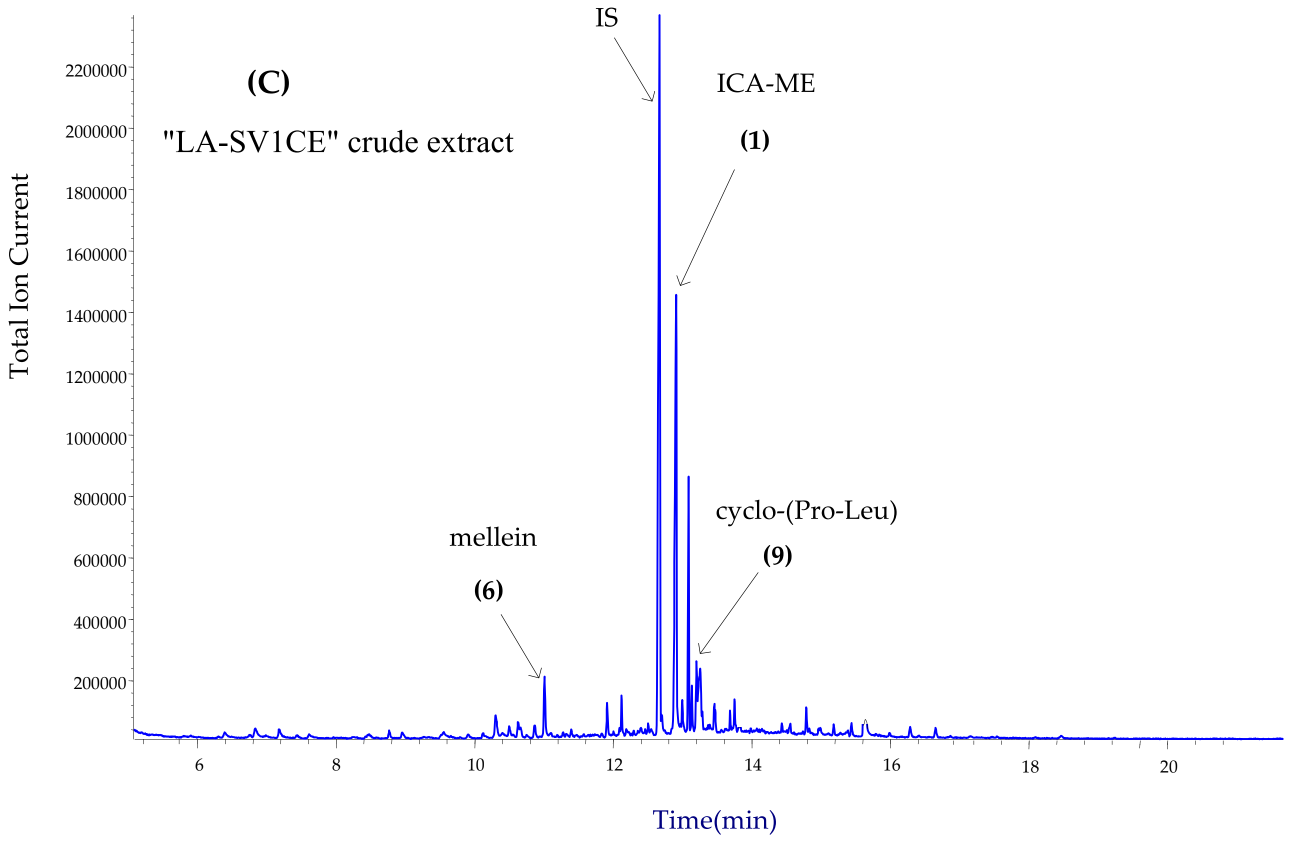

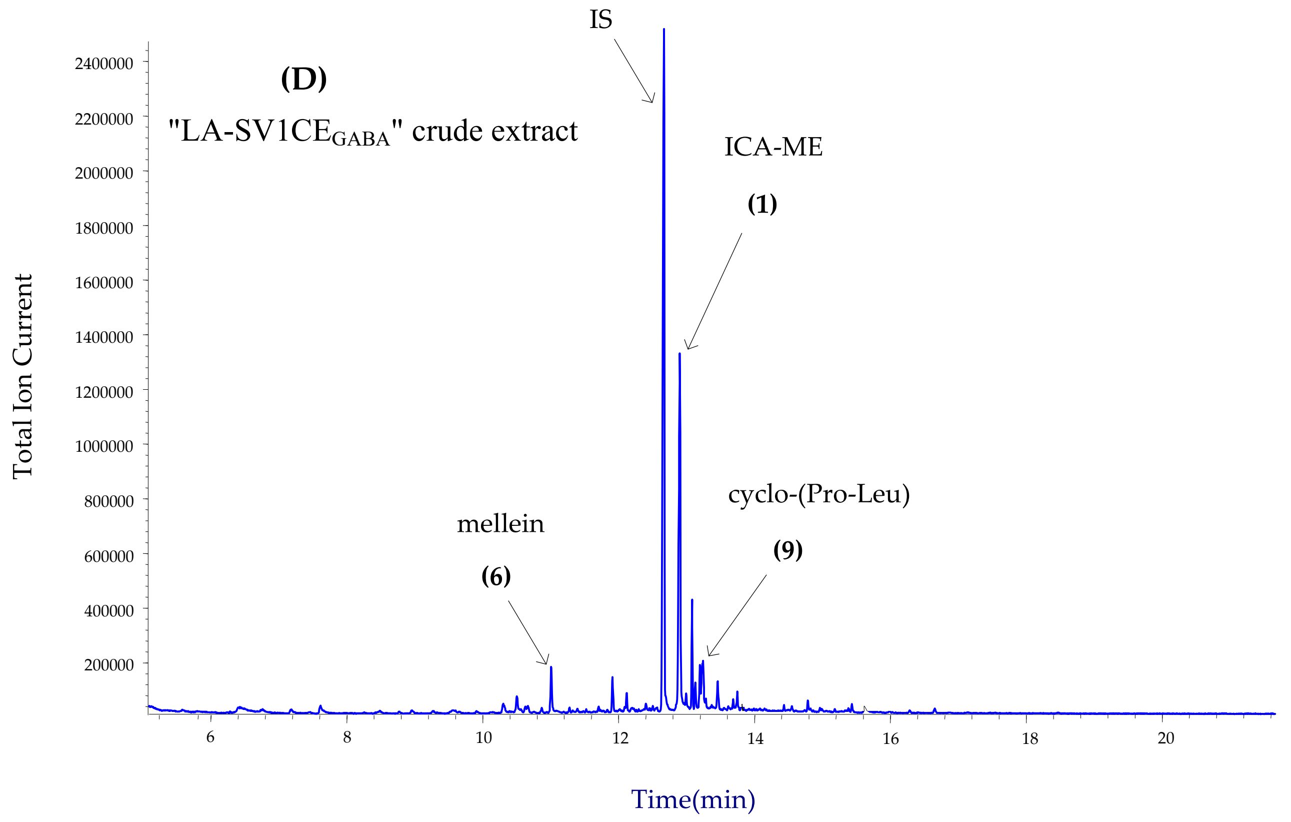

Figure S2. Annotated total ion chromatograms (TICs) acquired by processing crude extracts with diazomethane in ether. (C) Crude extract of *L. theobromae* LA-SV1(LA-SV1CE); (D) crude extract of *L. theobromae* LA-SV1 grown in presence of GABA (LA-SV1CE$_{GABA}$).

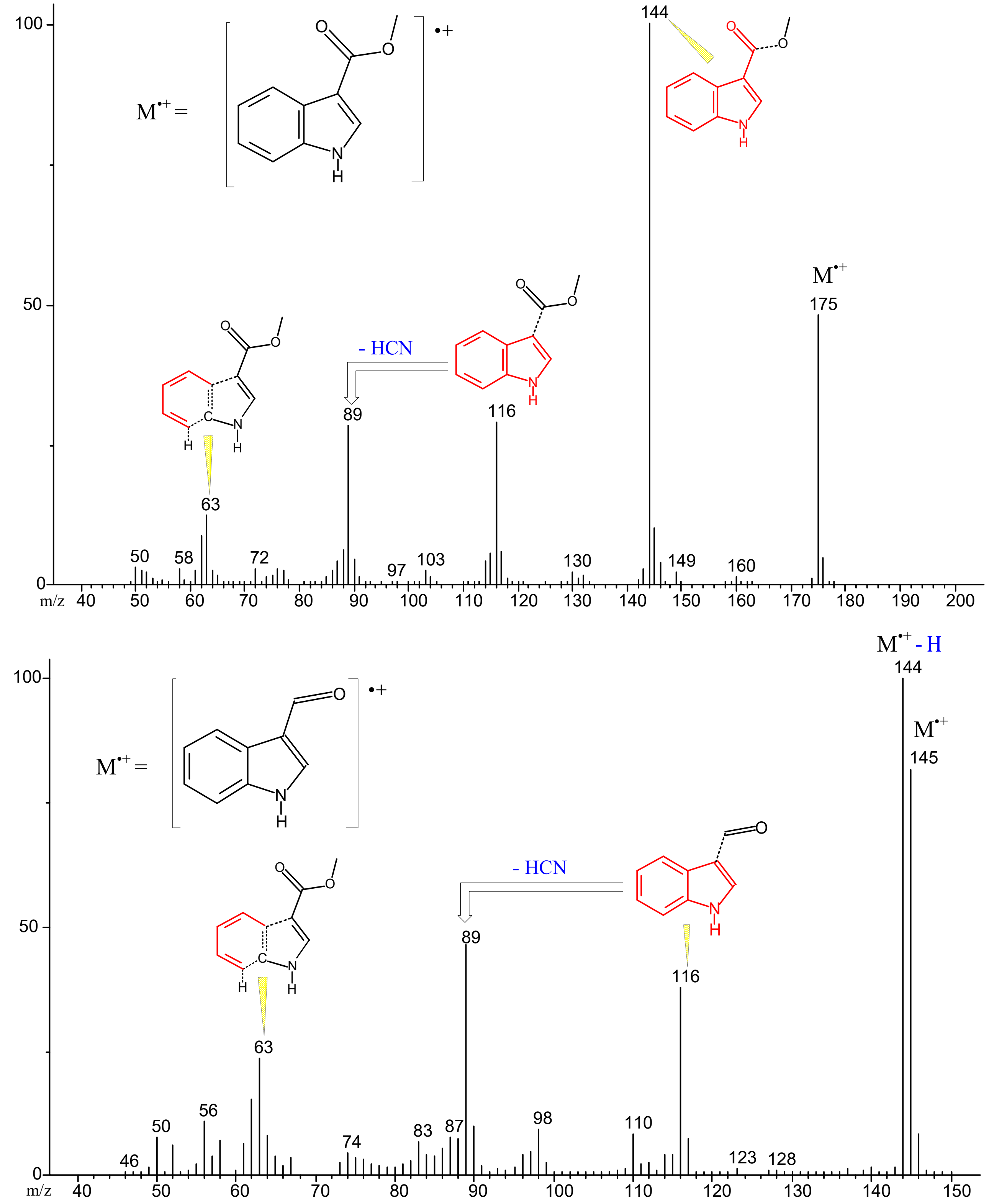

Figure S3. Annotated 70 eV EI mass spectra of **(1)** Indole-3-carboxylic acid methyl ester (Kovats RI = 1535) and **(2)** Indolecarboxaldehyde (Kovats RI = 1452)

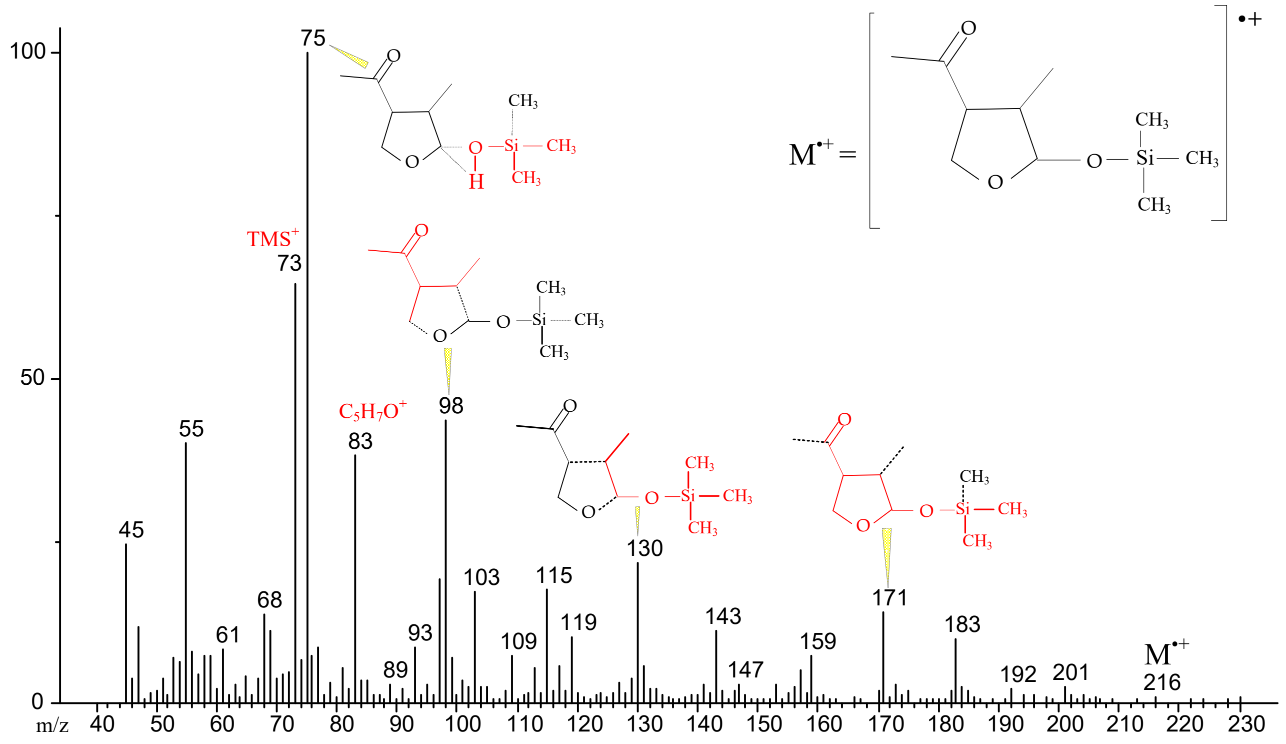

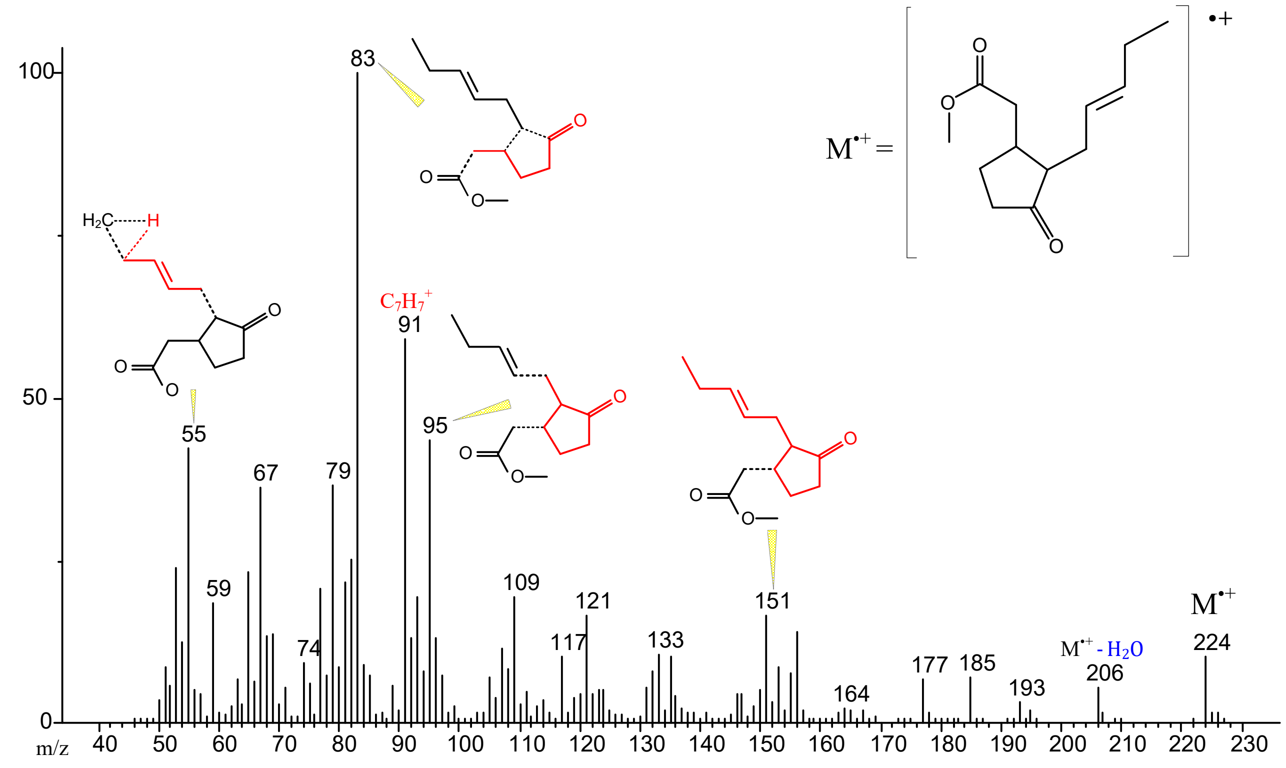

Figure S4. Annotated 70 eV EI mass spectra of (**3**) Botryodiplodin•TMS (Kovats RI = 1282) and (**4**) Jasmonic acid methyl ester (Kovats RI = 1598).

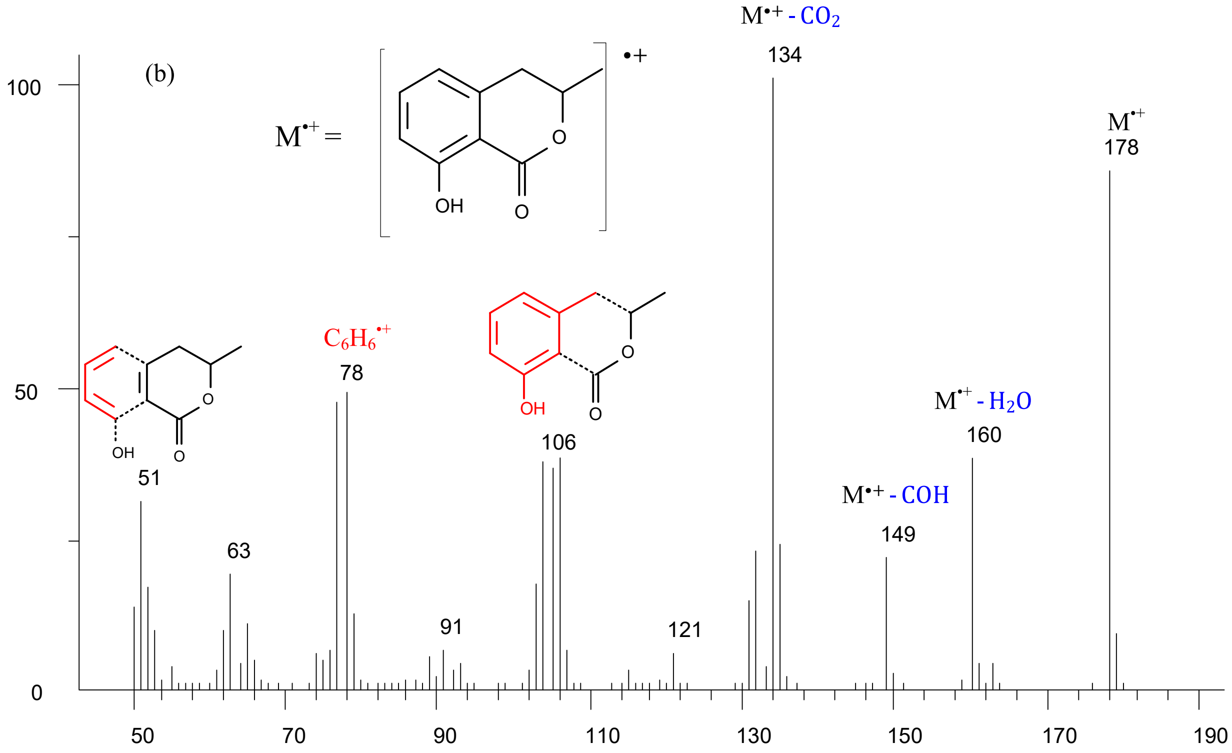

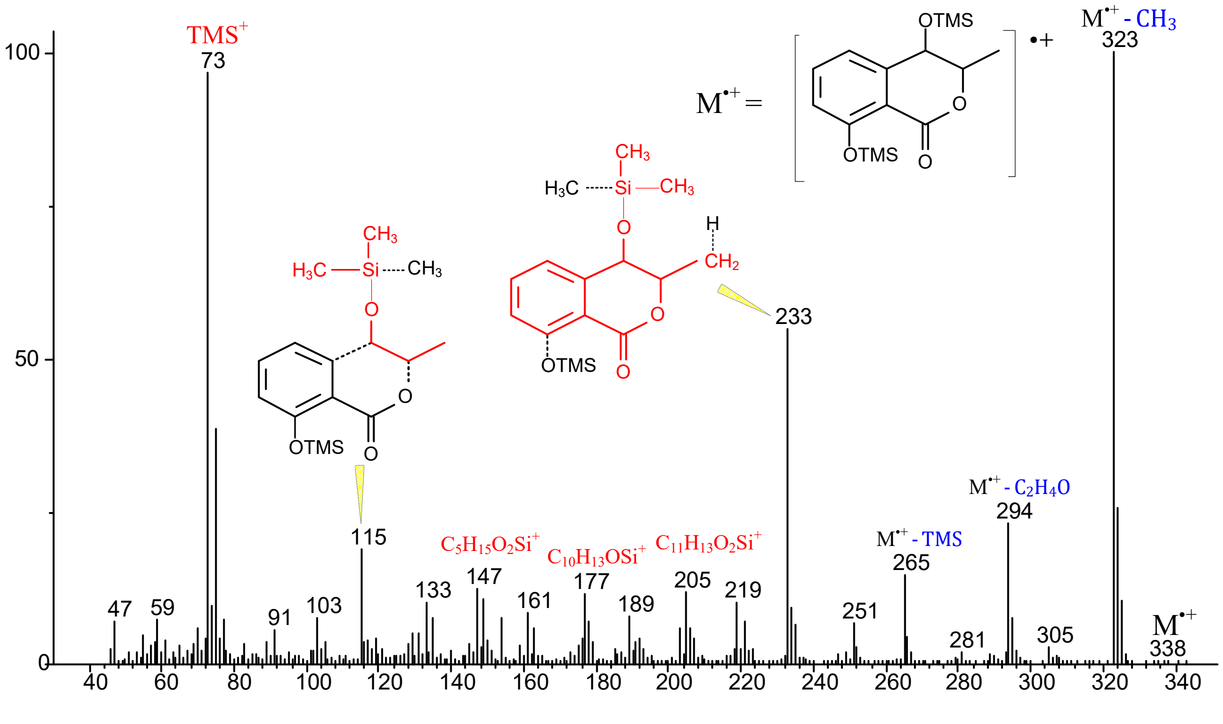

Figure S5. Annotated 70 eV EI mass spectra of Mellein (Kovats RI = 1656) and Hydroxymellein•2TMS (Kovats RI = 1605)

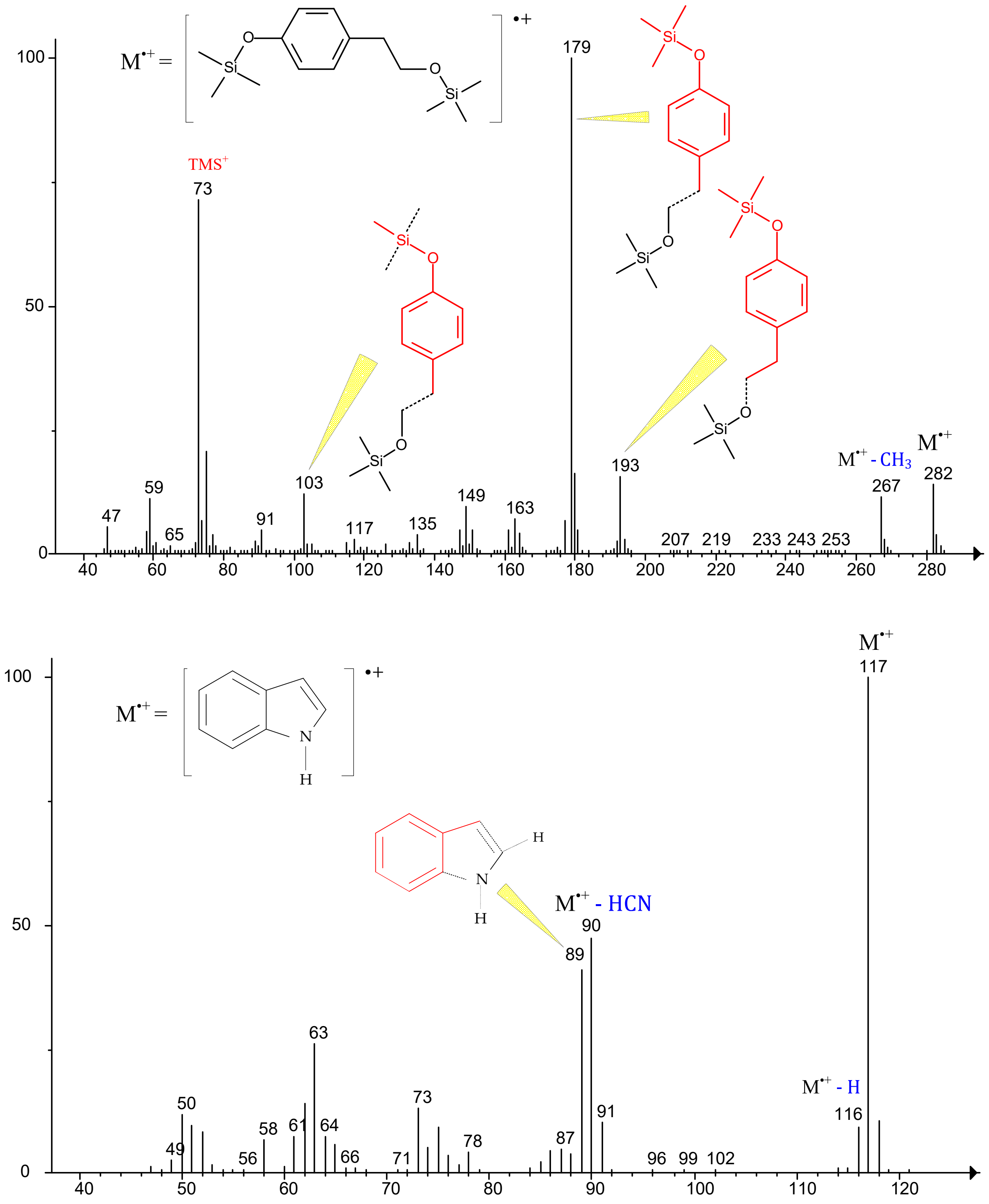

Figure S6. Annotated 70 eV EI mass spectra of **(7)** Tyrosol•2TMS (Kovats RI = 1575) and **(8)** Indole (Kovats RI = 1172)

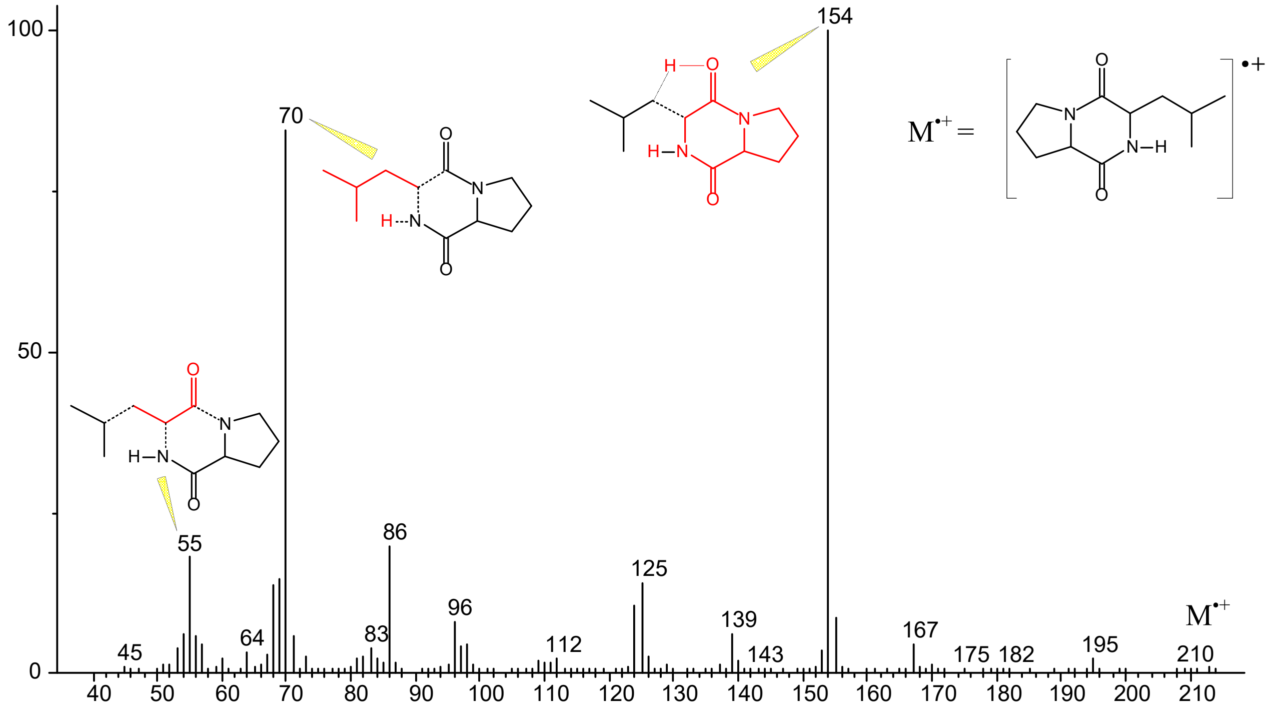

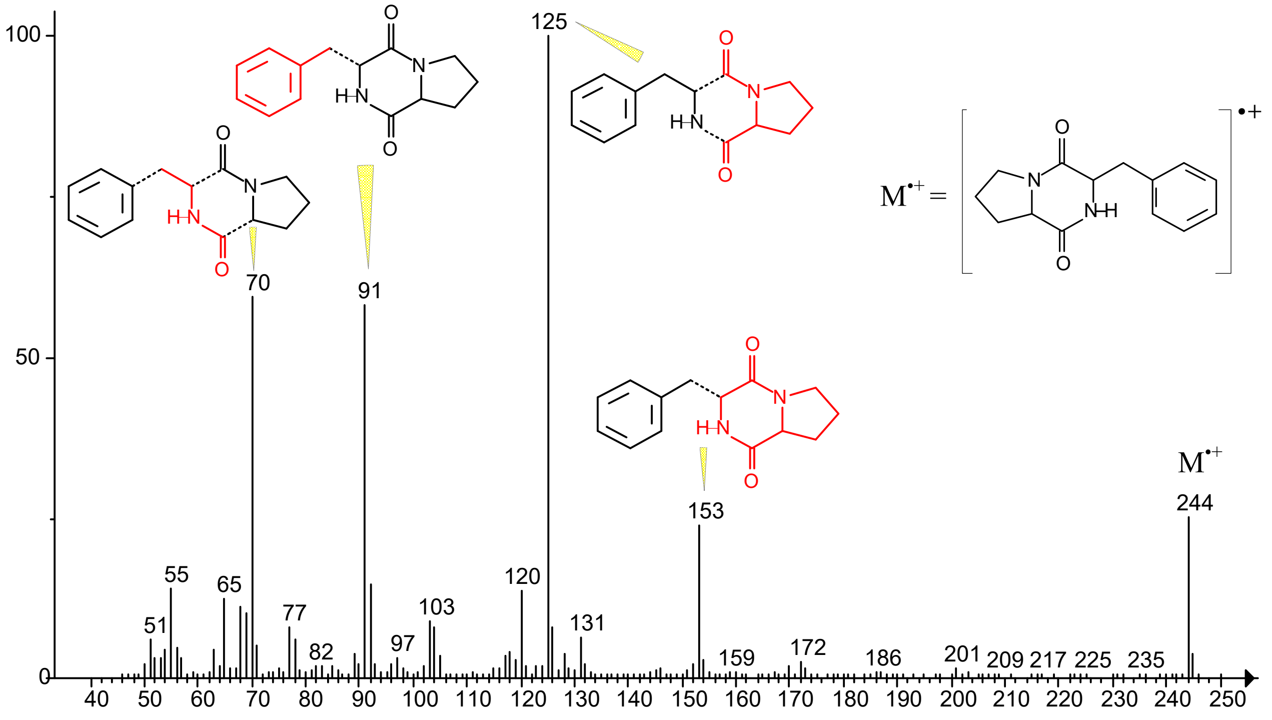

Figure S7. Annotated 70 eV EI mass spectra of **(9)** *Cyclo*-(Pro-Leu) (Kovats RI = 2068) and **(10)** *Cyclo*-(Pro-Phe) (Kovats RI = 2443).

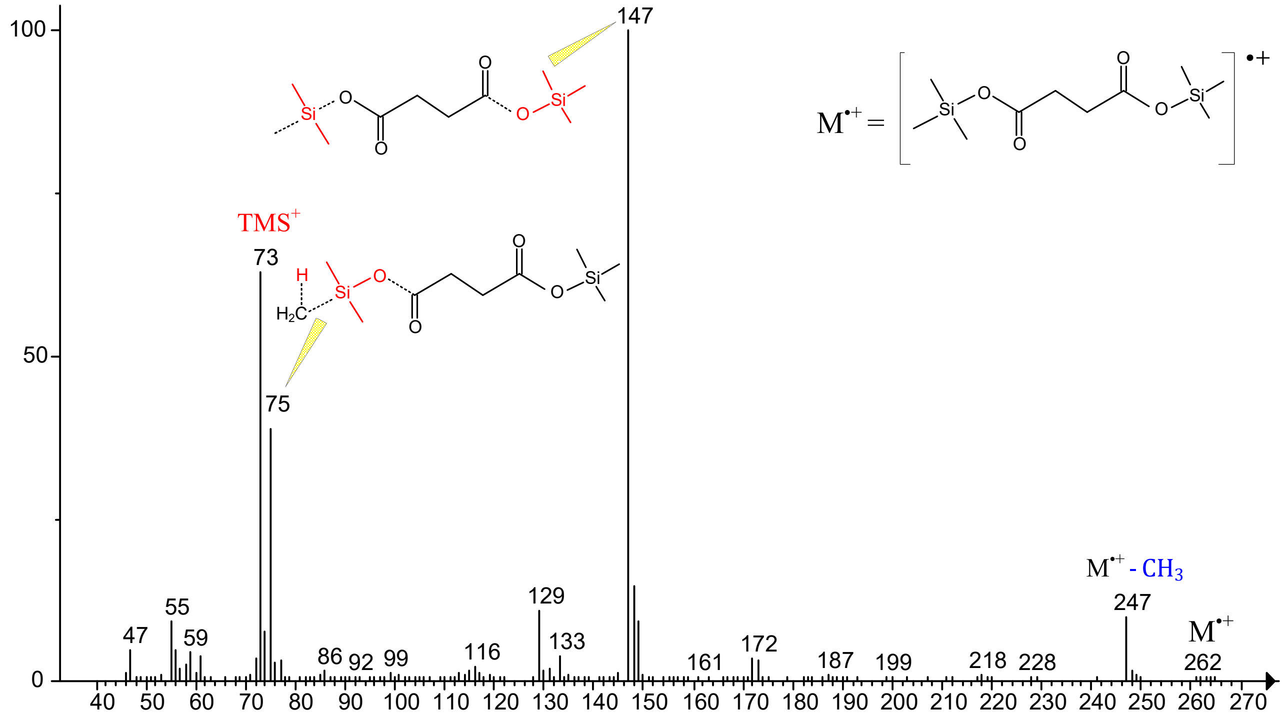

Figure S8. Annotated 70 eV EI mass spectra of **(11)** Succinic acid •2TMS (Kovats RI = 1310).

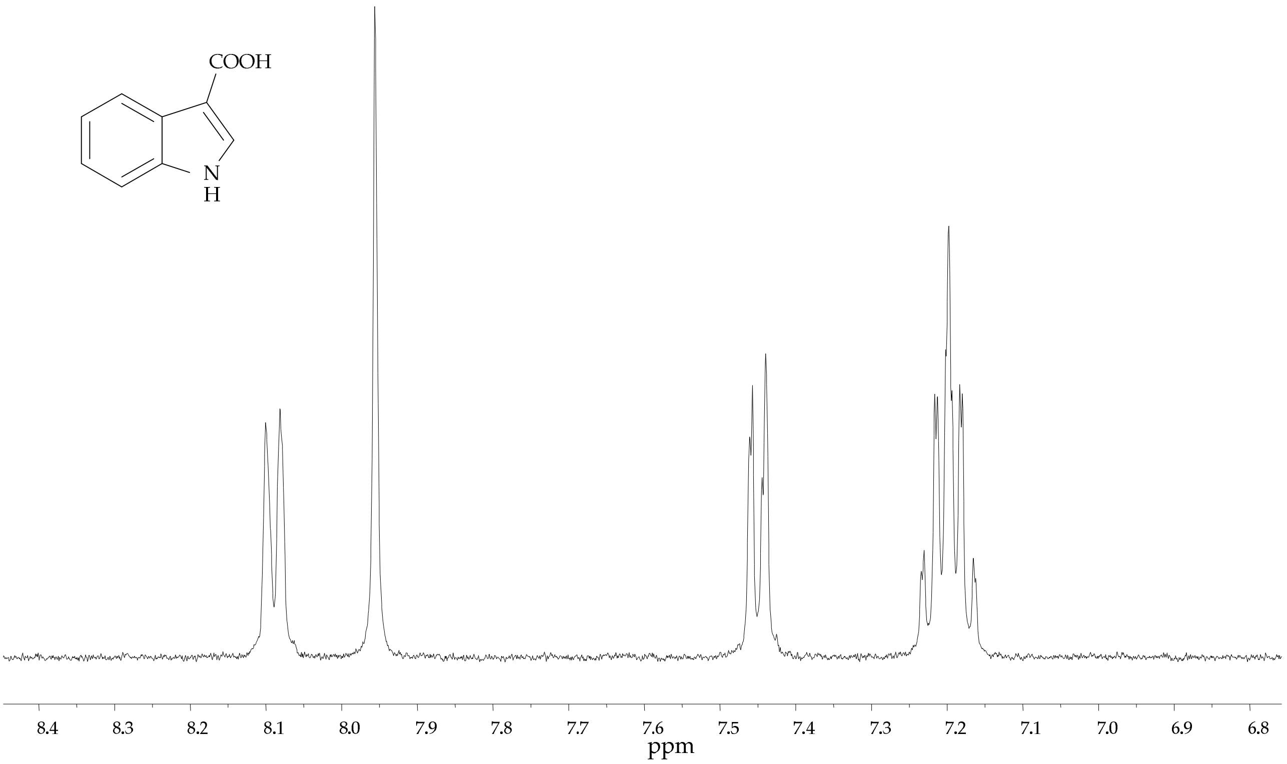

Figure S9. ^{1}H NMR spectrum of 3-indol-carboxylic acid (**1**) recorded at 400 MHz in MeOD.

(3R,4S)-Botryodiplodin **(3).**

[α]$_{25}$^{D} -62 (c 0.3, $CHCl_3$).

^{1}H NMR ($CDCl_3$, 400 MHz)

δ: 5.22-5.18 (m, H-2 and H-2′),
4.86 (d, *J*=12.0, OH′),
4.31 (t, *J*=8.8, H5),
4.12-4.02 (m, H5 and H5′),
3.69 (q, *J*=7.1, H4′),
3.44 (dt, *J*=2.7, 7.7, H4),
2.63 (quint, *J*=7.1, H3),
2.51-2.46 (m, H3′),
2.32 (s, H3-8),
2.23 (s, H3-8′),
1.09 (d, *J*=7.2, H3-6),
0.89 (d, *J*=7.2, H3-6).

Optical rotation and ^{1}H NMR spectrum were similar to data previously reported [35]

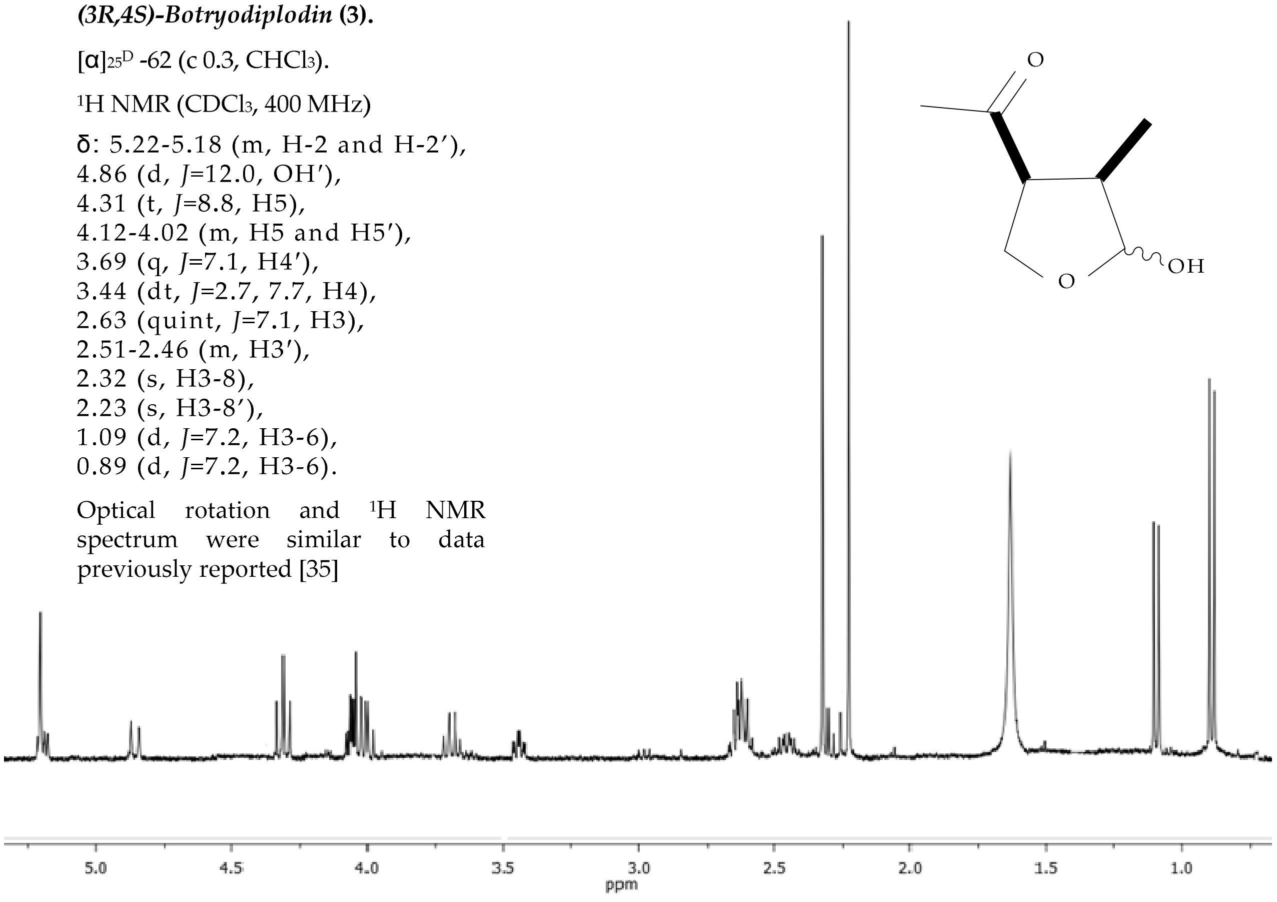

Figure S10. ^{1}H NMR spectrum of (-)-botryodiplodin (**3**) recorded at 400 MHz in $CDCl_3$

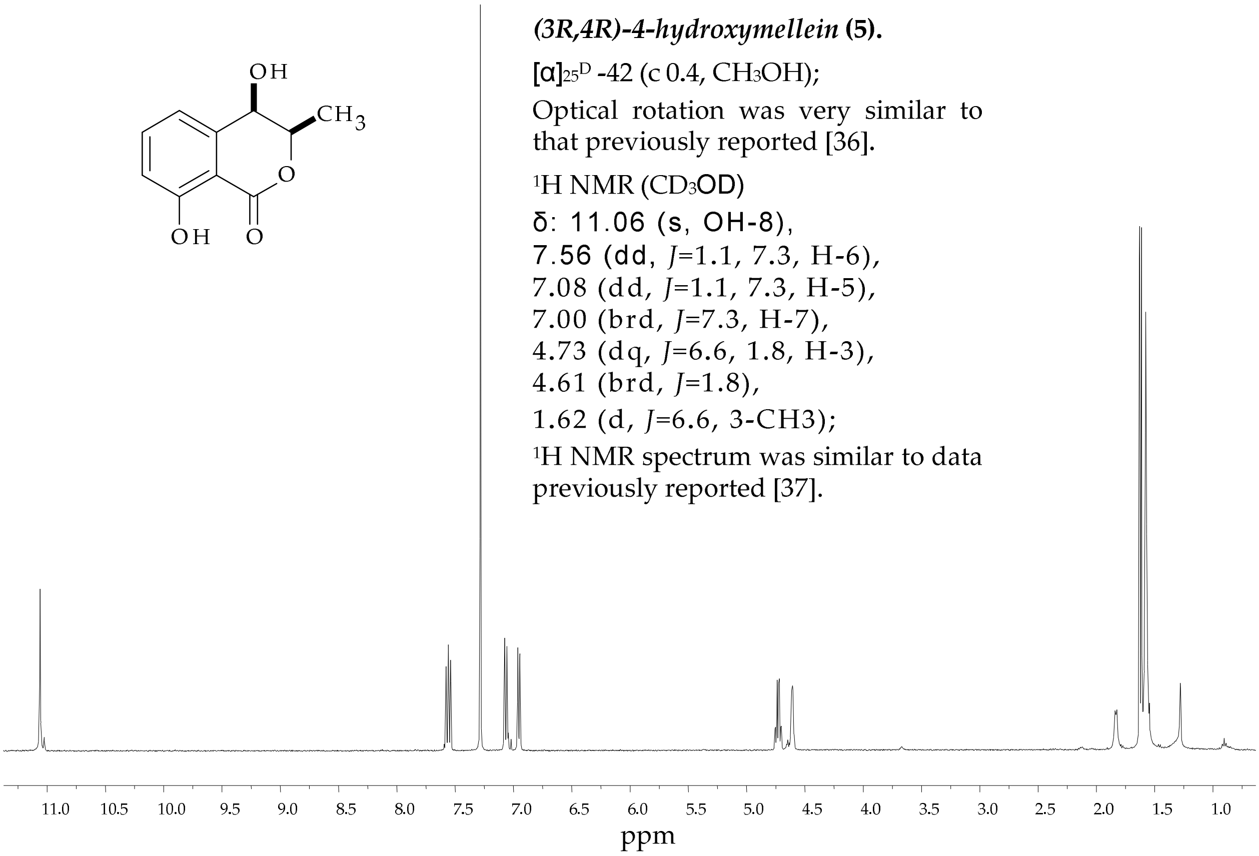

(3R,4R)-4-hydroxymellein **(5).**

$[\alpha]_{25}^{D}$ -42 (c 0.4, CH_3OH);

Optical rotation was very similar to that previously reported [36].

^{1}H NMR (CD_3OD)

δ: 11.06 (s, OH-8),
7.56 (dd, *J*=1.1, 7.3, H-6),
7.08 (dd, *J*=1.1, 7.3, H-5),
7.00 (brd, *J*=7.3, H-7),
4.73 (dq, *J*=6.6, 1.8, H-3),
4.61 (brd, *J*=1.8),
1.62 (d, *J*=6.6, 3-CH3);

^{1}H NMR spectrum was similar to data previously reported [37].

Figure S11. ^{1}H NMR spectrum of (3*R*,4*R*)-4-hydoxymellein (**5**) recorded at 400 MHz in $CDCl_3$

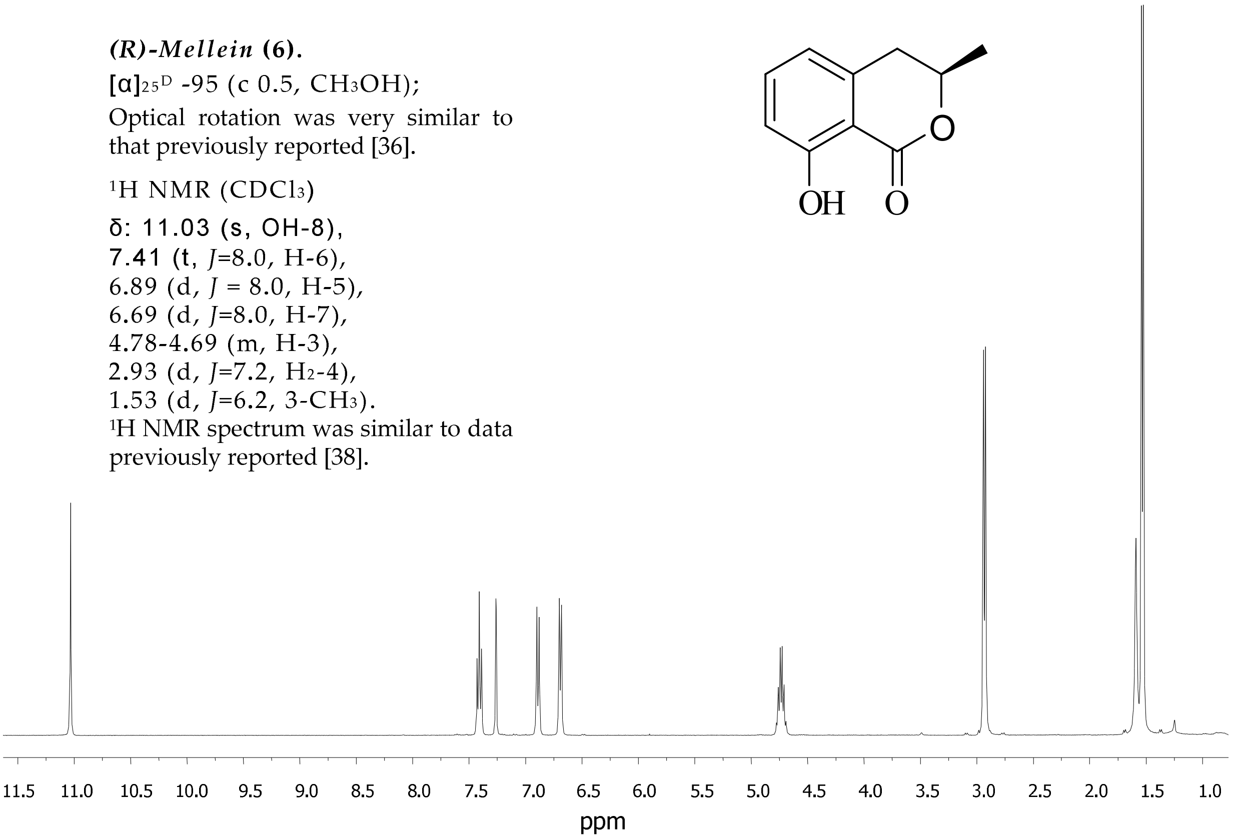

(R)-Mellein **(6).**

$[\alpha]_{25}^{D}$ -95 (c 0.5, CH_3OH);

Optical rotation was very similar to that previously reported [36].

^{1}H NMR ($CDCl_3$)

δ: 11.03 (s, OH-8),
7.41 (t, *J*=8.0, H-6),
6.89 (d, *J* = 8.0, H-5),
6.69 (d, *J*=8.0, H-7),
4.78-4.69 (m, H-3),
2.93 (d, *J*=7.2, H_2-4),
1.53 (d, *J*=6.2, 3-CH_3).

^{1}H NMR spectrum was similar to data previously reported [38].

Figure S12. ^{1}H NMR spectrum of (*R*)-mellein (**6**) recorded at 400 MHz in $CDCl_3$
